# Size and charge effect of guest cations in the formation of polyoxopalladates: a theoretical and experimental study†
†Dedicated to Professor Walter Klemperer on the occasion of his 70^th^ birthday.
‡
‡Electronic supplementary information (ESI) available: Details on experimental methods; the experimental and computational related tables and figures. CCDC 1555480–1555483. For ESI and crystallographic data in CIF or other electronic format see DOI: 10.1039/c7sc03441e



**DOI:** 10.1039/c7sc03441e

**Published:** 2017-09-25

**Authors:** Zhongling Lang, Peng Yang, Zhengguo Lin, Likai Yan, Ming-Xing Li, Jorge J. Carbó, Ulrich Kortz, Josep M. Poblet

**Affiliations:** a Departament de Química Física i Inorgànica , Universitat Rovira i Virgili , c/Marcel lí Domingo 1 , 43007 Tarragona , Spain . Email: josepmaria.poblet@urv.cat; b Department of Life Sciences and Chemistry , Jacobs University , Campus Ring 1 , 28759 Bremen , Germany . Email: u.kortz@jacobs-university.de; c Institute of Functional Material Chemistry , Faculty of Chemistry , Northeast Normal University , Changchun 130024 , P. R. China; d Department of Chemistry , College of Sciences , Shanghai University , Shanghai 200444 , P. R. China

## Abstract

The development of rational synthetic procedures with desired nuclearity and high selectivity is a critical issue in inorganic chemistry. Here we demonstrate a comprehensive understanding of the template effect induced by metal cations in the formation mechanism of the class of polyoxopalladates ({MPd_12_L_8_} nanocube and {MPd_15_L_10_} nanostar) by combining computational and experimental techniques. The capture of a M^*n*+^ guest ion by a peripheral palladium(ii)-oxo shell leads to a competition between the parent Pd^2+^ addenda ion and the respective guest metal ion. The present study reveals that (i) the selection of the incorporated guest ion has a thermodynamic control, (ii) the main factors governing the formation of a particular polyanion are the charge and size of the guest cation, (iii) the electrostatic interaction between the cation and the surrounding oxo ligands and (iv) the dehydration ability of the cation. As expected from the number of observed {M^*n*+^Pd_12_L_8_} species, trivalent cations M^3+^ were found to be good templates resulting in several examples of {M^3+^Pd_12_L_8_}, whereas monovalent cations M^+^ are much less prone to form {M^+^Pd_12_L_8_}. For tetravalent cations the dehydration energies are very large, however, the formation of {M^4+^Pd_12_L_8_} nanocubes is found to be still energetic favourable. Fully consistent with computational predictions, four novel polyoxo-12-palladates were synthesized: the La^3+^-centered nanocube [LaPd_12_O_8_(PhAsO_3_)_8_]^5–^ (**LaPd_12_-closed**), the La^3+^-centered “open” nanocube [LaPd_12_O_6_(OH)_3_(PhAsO_3_)_6_(OAc)_3_]^3–^ (**LaPd_12_-open**), the Ga^3+^-centered [GaPd_12_O_8_(PhAsO_3_)_8_]^5–^ (**GaPd_12_**), and the In^3+^-analogue [InPd_12_O_8_(PhAsO_3_)_8_]^5–^ (**InPd_12_**). All four compounds were fully characterized in the solid state and in solution by a multitude of physicochemical techniques, including ^71^Ga and ^115^In NMR as well as mass spectrometry. The experimentally observed selective incorporation of only In^3+^ ions in the presence of Ga^3+^ and In^3+^ confirmed the thermodynamic control of the formation mechanism, which we had predicted by theory.

## Abstract 2

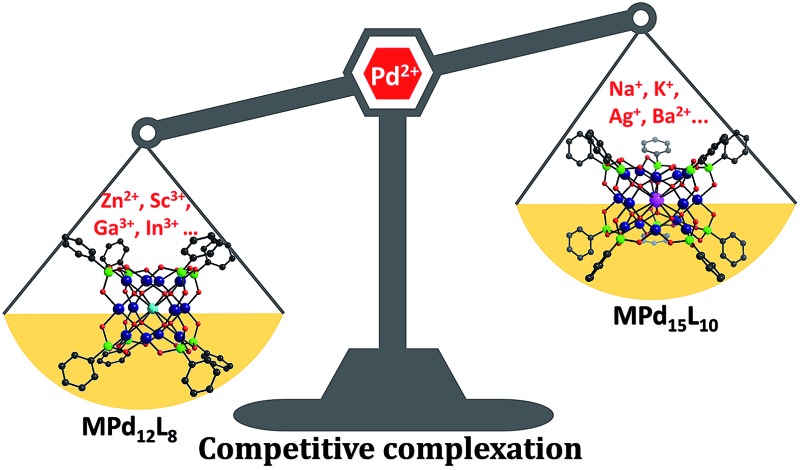
The close interplay of theoretical and experimental techniques can facilitate the understanding and rational synthesis of novel inorganic clusters, and here an impressive example is shown for the class of cuboid-shaped polyoxo-12-palladates(ii) with a central metal ion guest.

## 1. Introduction

In recent years significant advances have been made in the control of different variables involved in the formation mechanism of polyoxometalates (POMs). However, there is a lack of systematic studies that aim at identifying unambiguously the driving forces related to the self-assembly or aggregation processes. The “template-directed” method is one popular and extremely important synthetic strategy for obtaining novel POMs. The nuclearity and topology of the products are strongly dependent on the size, shape, and charge of the template ions. For example, anions such as Cl^–^ and SO_4_^2–^ could be encapsulated in the central cavities of discrete polyoxovanadates to form templated host–guest complexes.^1^ On the other hand, many different heterogroups (*e.g.*, As^III^O_3_^3–^, Sb^III^O_3_^3–^, Si^IV^O_4_^4–^, Ge^IV^O_4_^4–^, P^V^O_4_^3–^, As^V^O_4_^3–^, and I^VII^O_6_^5–^) can efficiently control the shape, size, and structure of high-nuclearity heteropolytungstates.^2^

Besides classical POMs,^3^ in the last decade or so an “unconventional” POM family based exclusively on Pd^II^, Pt^III^, or Au^III^ addenda has been developed.^4^ Since noble metals are well-known active ingredients of many catalysts, the study of noble metal-containing POMs is a particularly interesting topic. In 2004, Wickleder's group synthesized the first polyoxoplatinate exclusively based on d^7^ addenda ions, [Pt^III^_12_O_8_(S^VI^O_4_)_12_]^4–^.^5^ Since then the Kortz group has pioneered the class of polyoxopalladates(ii) in 2008,^6^ and the class of polyoxoaurates(iii) in 2010.^7*a*^ The first polyoxopalladate was the [H_6_Pd_13_O_8_(AsO_4_)_8_]^8–^ (**Pd_13_**) nanocube, prepared by simple condensation of Pd^2+^ and arsenate (As^V^O_4_^3–^) ions in aqueous medium.^6^ In the following years it was demonstrated that the eight arsenate capping groups can be easily replaced by other heterogroups, such as selenite ([Pd_13_O_8_(SeO_3_)_8_]^6–^, **Pd_13_Se**) or phenylarsonate ([Pd_13_O_8_(PhAsO_3_)_8_]^6–^, **Pd_13_AsPh**).^8^ Interestingly, substitution of the capping groups is accompanied by an increase of the coordination number of the central Pd^2+^ ion from 4 (**Pd_13_**) to 6 (**Pd_13_Se**) and even 8 (**Pd_13_AsPh**).

**Fig. 1:**
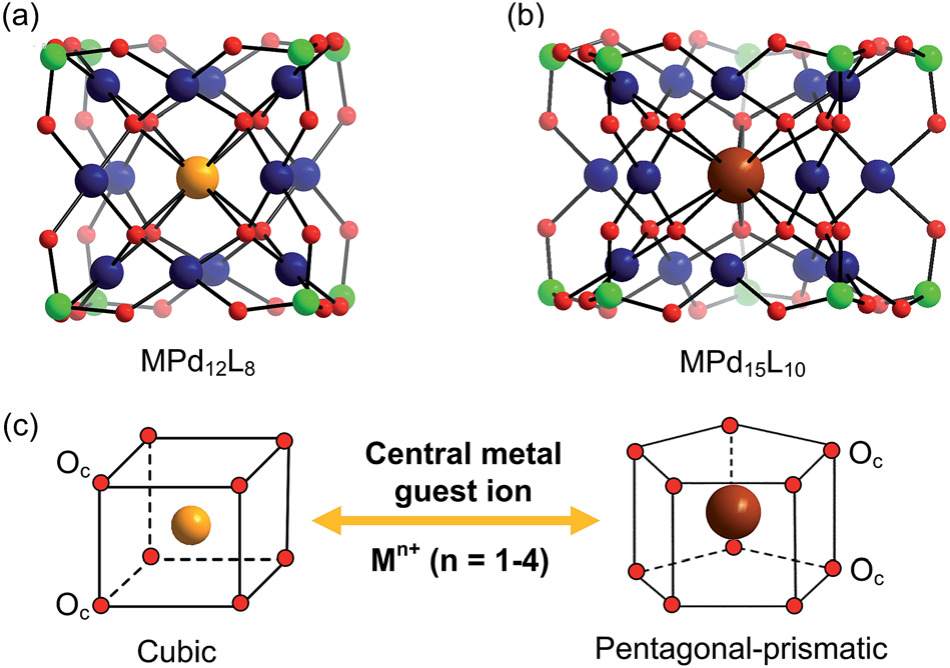
Ball-and-stick representation of the polyoxopalladate (a) nanocube {MPd_12_L_8_} and (b) nanostar {MPd_15_L_10_}, respectively (M = central metal guest ion, L = capping group), and (c) the oxo-coordination sphere around M in both palladate structural types. Colour code: M (orange/brown), L (green), Pd (dark blue), O (red).

In addition to the capping groups the central palladium(ii) ion in the nanocube {MPd_12_L_8_} (Fig. 1a) can also be replaced by other metal ion guests, including trivalent lanthanide ions (Ln^3+^ = Y, Pr, Nd, Sm, Eu, Gd, Tb, Dy, Ho, Er, Tm, Yb, Lu) and 3d transition metal ions (Sc^3+^, Mn^2+^, Fe^3+^, Co^2+^, Ni^2+^, Cu^2+^, Zn^2+^).^9^ Interestingly, the nanostar {MPd_15_L_10_} (Fig. 1b) can be formed only in the presence of Na^+^, K^+^, Ag^+^, or Ba^2+^,^10^ whereas in the presence of Sr^2+^ ions the “open” nanocube {SrPd_12_L_6_L′_3_} (L = phenylarsonate, L′ = acetate) is formed.^10*e*^ Such observations bear similarities with the important template role of alkali and alkaline earth ions in the formation of various organic macrocycle-based structures (crown ethers *etc.*).^11^ In addition to the above-mentioned nanocube, nanostar and open-nanocube structural types, some additional polyoxopalladates with unexpected geometries have been obtained, such as the bowl-shaped palladovanadate {Pd_7_V_6_},^12*a*^ the double cuboid-shaped 22-palladates {Cu_2_Pd_22_}^12*b*^ and {Na_2_Pd_22_},^12*c*^ as well as palladate macrocycles {Pd_*n*_} (*n* = 60, 72, 84, 96, 108).^13^

It is evident that the central metal ion guest as well as the capping groups play a key role in the formation mechanism of polyoxopalladates, but details remain unknown. Hence the rational synthesis of novel polyoxopalladate structural types of desired shape, size and composition is virtually impossible. On the other hand, density functional theory (DFT) methods have been applied to POMs significantly in the last two decades, in particular with respect to (i) electronic structure, (ii) rationalization of physicochemical properties, and (iii) reactivity as a function of shape and composition.^14^ In order to obtain more insight into polyoxopalladate chemistry, in particular with respect to factors that govern guest metal ion encapsulation and to perhaps shed more light on selectivity issues, we have decided to perform systematic theoretical analysis for a series of 35 metal ion guests M involved in the formation of the {MPd_12_L_8_} nanocube and {MPd_15_L_10_} nanostar polyoxopalladate structural types.

The encapsulated cations were selected by considering both their charge and size, which range from alkali and alkaline earth ions to transition metal ions, as well as trivalent and tetravalent main group cations. We have discovered a remarkable competition between Pd^2+^ ions and other cations, which is key for the formation of a specific polyoxopalladate structural type. With a focus on eventually being able to computationally predict experimental results, we have carefully studied experimentally (i) the capture of the largest trivalent cation La^3+^ inside a polyoxopalladate, and (ii) the selective incorporation of In^3+^ *vs.* Ga^3+^ in a polyoxopalladate.

## 2. Computational and experimental details

### 2.1 Computational method

All calculations were performed with the Gaussian 09 package.^15^ The computational scheme consists of two steps. Geometry optimizations of all nanocube {MPd_12_L_8_} and nanostar {MPd_15_L_10_} polyoxopalladates were carried out at the B3LYP level without symmetry restrictions.^16^ The SDD effective core pseudopotential (ECP) basis set was used for La, Yb, Lu, Eu, Ce, Th, and U,^17^ the small core potential (CRENBL ECP) was used for Ra,^18^ whereas the LANL2DZ basis set was employed for the main group metals (Rb, Cs, Sr, Ba, Ga, In, Tl, Sn) and transition metals (Sc, Mn, Fe, Co, Ni, Cu, Zn, Y, Zr, Pd, Ag, Cd, Hf) with Los Alamos relativistic core potentials (ECPs).^19^ In addition, the 6-31G** basis set was used for O, C, As, Se, H, and the encapsulated small metal ions (Li, Na, K, Be, Mg, Ca).^20^ From these calculations, we obtained the energies at the B3LYP level. In order to include the long-range interaction and dispersion effects, single point calculations were performed for all optimized polyanions with two additional functionals, M06 and ωB97XD.^21,22^ For all steps, the continuum SMD implicit solvation model was used to simulate the effect of the aqueous solution.^23^

As shown in Fig. 1, the nanocube {MPd_12_L_8_} and nanostar {MPd_15_L_10_} were selected, in which the central metal ion usually has an oxo-coordination number of 8 and 10, respectively. In order to evaluate the selective encapsulation of different guest metal ions, the reaction mechanism is simulated by scheme (1), and the complexation energy (*E*_com_) was calculated by eqn (2) and (3):1
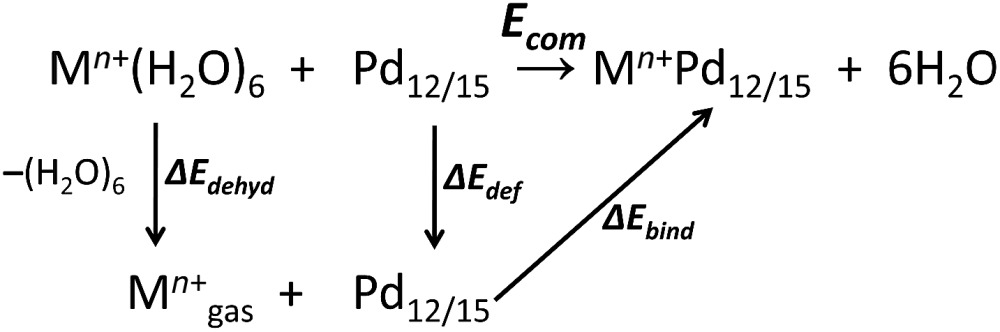

2 *E*_com_ = *E*(M^*n*+^Pd_12/15_) + *E*(6H_2_O) – *E*(**Pd**_**12/15**_) – *E*(M^*n*+^(H_2_O)_6_)

3 *E*_com_ = Δ*E*_bind_ + Δ*E*_def_ + Δ*E*_dehyd_

where M^*n*+^(H_2_O)_6_ is the solvated cation model. Since dehydration is one fundamental factor for an accurate determination of the complexation energy, all cations were solvated by six explicit water molecules and also surrounding by the implicit model. With this explicit and implicit solvent combination model, the dehydration energy of the cations was well estimated when compared with the experimental hydration enthalpies, as all deviations are <10%, except for Li^+^, Rb^+^, Cs^+^, and Ra^2+^ (Fig. S1‡).^24^ The entire polyoxopalladate formation process is completed when the relaxed **Pd_12/15_** cage picks up the cation from the solvent and encapsulates it into the cage. The complexation energies (*E*_com_) for **Pd_12/15_** with all different cations are computed, defined as the reaction energies involved in the encapsulation process. Three terms contribute to this process, (i) the cage deformation of each capsule with respect to the cation-free **Pd_12/15_** cage (Δ*E*_def_), (ii) dehydration of the guest cation from solvated to free ion (Δ*E*_dehyd_), and (iii) the binding interaction (Δ*E*_bind_) between the optimally-deformed cage and the “naked” cation, respectively.

### 2.2 Synthesis of Na_5_[LaPd_12_O_8_(PhAsO_3_)_8_]·31H_2_O (**Na-LaPd_12_-closed**)

Pd(CH_3_COO)_2_ (0.023 g, 0.102 mmol), PhAsO_3_H_2_ (0.020 g, 0.099 mmol) and LaCl_3_·7H_2_O (0.009 g, 0.024 mmol) were dissolved in 2 mL of 0.5 M NaOAc solution (pH 7.0). While stirring, the solution was heated to 80 °C. After dissolution of all components, the pH of the reaction mixture was adjusted to 7.3 by addition of 6 M NaOH. The resulting solution was heated at 80 °C for 1 hour. Then it was cooled to room temperature, filtered and allowed to crystallize at room temperature in an open vial. Dark red, block-shaped crystals of **Na-LaPd_12_-closed** were obtained after one week, which were filtered off and air dried. Yield: 0.008 g (25%, based on Pd). Elemental analysis calcd (%): Na 3.01, C 15.10, La 3.64, Pd 33.45, As 15.70; found: Na 3.28, C 15.51, La 3.76, Pd 33.44, As 15.43. IR (2% KBr pellet, *ν*/cm^–1^): 1627 (m), 1479 (w), 1439 (m), 1093 (m), 792 (s), 742 (w), 694 (m), 592 (m), 547 (s).

### 2.3 Synthesis of Na_3_[LaPd_12_O_6_(OH)_3_(PhAsO_3_)_6_(OAc)_3_]·40H_2_O (**Na-LaPd_12_-open**)

This compound was prepared by exactly the same procedure as **Na-LaPd_12_-closed**. The initially formed **Na-LaPd_12_-closed** was removed by filtration. The filtrate was left for further evaporation, which resulted in another portion of dark red, needle-like crystals of **Na-LaPd_12_-open** within a few days, which were filtered off and air dried. Yield: 0.006 g (19% based on Pd). Elemental analysis calcd (%): Na 1.85, C 13.52, La 3.72, Pd 34.24, As 12.05; found: Na 1.94, C 13.75, La 3.51, Pd 34.69, As 12.66. IR (2% KBr pellet, *ν*/cm^–1^): 1633 (m), 1539 (m), 1439 (w), 1419 (m), 1093 (m), 814 (s), 744 (m), 694 (m), 536 (s).

### 2.4 Synthesis of Na_5_[GaPd_12_O_8_(PhAsO_3_)_8_]·36H_2_O (**Na-GaPd_12_**)

Pd(CH_3_COO)_2_ (0.023 g, 0.102 mmol), PhAsO_3_H_2_ (0.020 g, 0.099 mmol) and Ga(NO_3_)_3_ (0.006 g, 0.024 mmol) were dissolved in 2 mL of 0.5 M NaOAc solution (pH 7.0). While stirring, the solution was heated to 80 °C for 1 hour. Then it was cooled to room temperature, filtered and allowed to crystallize at room temperature in an open vial. Red, block-shaped crystals of **Na-GaPd_12_** were obtained after three days, which were filtered off and air dried. Yield: 0.014 g (43% based on Pd). Elemental analysis calcd (%): Na 2.99, C 15.02, Ga 1.82, Pd 33.27, As 15.62; found: Na 2.90, C 15.71, Ga 1.96, Pd 32.34, As 16.14. IR (2% KBr pellet, *ν*/cm^–1^): 1631 (m), 1479 (w), 1438 (m), 1094 (m), 804 (s), 744 (w), 694 (m), 657 (m), 538 (s).

### 2.5 Synthesis of Na_5_[InPd_12_O_8_(PhAsO_3_)_8_]·30H_2_O (**Na-InPd_12_**)

This compound was prepared by exactly the same procedure as **Na-GaPd_12_**, but with InCl_3_ (0.005 g, 0.025 mmol) instead of Ga(NO_3_)_3_. Red, block-shaped crystals of **Na-InPd_12_** were obtained after three days, which were filtered off and air dried. Yield: 0.014 g (44% based on Pd). Elemental analysis calcd (%): Na 3.04, C 15.27, In 3.04, Pd 33.22, As 15.88; found: Na 2.82, C 15.89, In 3.80, Pd 32.30, As 16.06. IR (2% KBr pellet, *ν*/cm^–1^): 1628 (m), 1479 (w), 1439 (m), 1093 (m), 802 (s), 744 (w), 694 (m), 629 (m), 540 (s).

## 3. Results and discussion

### 3.1 Size matching between guest M^*n*+^ cations and the **Pd_12/15_** cages

Since the discovery in 2008 of the first member of the **Pd_12_** family, **Pd_13_**,^6^ Kortz's group has synthesized a large number (*ca.* 50) of palladate nanocube derivatives {MPd_12_L_8_} by systematically substituting the arsenate capping groups by selenite, phenylarsonate and phenylphosphonate, and the central Pd^2+^ ion by s, d and f block metal ions.^4,6–9^ In order to better understand the templating role of the guest cation, systematic DFT calculations were performed by introducing different metal ions into the **Pd_12_** and **Pd_15_** cages. The empty “host” cages **Pd_12_** and **Pd_15_** and the corresponding host (**Pd_12_/Pd_15_** cage) – guest (cation M) systems capped by PhAs^V^O_3_^2–^ ({MPd_12_(AsPh)_8_}) were optimized at the B3LYP level. The molecular electrostatic potential (MEP, Fig. S2‡) analysis for the free **Pd_12_** cage revealed an interesting distribution of most negative partial atomic charges at the centre of the inner {O_8_} cage, indicating that electrophiles such as metal ions are most likely to be located at the central position of the palladate nanocube. This prediction is in line with the experimental results, which show that the guest cation indeed occupies such position. As shown in Table S1,‡ the computed distances (O_c_–O_c,_ M–O_c_, and M–Pd) for a series of palladates reproduced fairly well the reported XRD data, although they are found systematically slightly longer than observed experimentally. It is worth mentioning that when M = Pd^2+^, Fe^3+^, Co^2+^, Ni^2+^, and Eu^3+^ ions occupy the central site, the DFT calculations suggested that the high-spin state for M in {MPd_12_(AsPh)_8_} is more favourable than the low-spin state. Such result was indeed confirmed experimentally by magnetic and EPR studies.^9*b*^

**Fig. 2:**
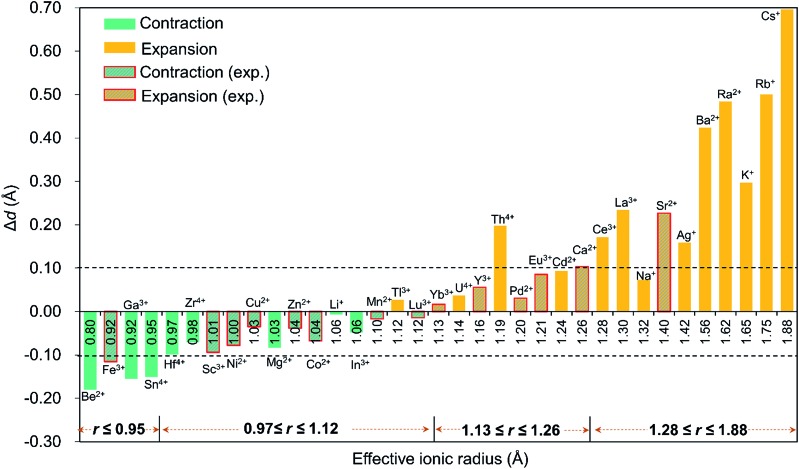
The O_c_–O_c_ distortions (Δ*d* = *d*_O–O_ – 2.728, with respect to the distance in the empty cage) of the {O_8_} cavity after encapsulation of the guest cation. The cations are organized from left to right according to their effective ionic radius. The columns with red edges represent palladates which have been prepared experimentally, whereas all other compounds have not been synthesized yet.

For efficient encapsulation, the guest cation and the cavity in the palladate cage should match well. Due to the small flexibility of the {O_8_} coordination shell, all guest cations are optimally located in the centre of the cube. The average O_c_–O_c_ distance generally elongates as the effective ionic radius of M^*n*+^ increases, and this trend is perfectly in line with an increase of the M–O_c_ bond distances. To gain insight into the effect of cation size on the geometry of the cage, the O_c_–O_c_ distortions after encapsulation of the cation are plotted in Fig. 2, by referring to the distance in the empty cage **Pd_12_** (*d*_O_c_–O_c__ = 2.728 Å). We found that guest cations with a radius smaller than 1.12 Å could induce a contraction of the {O_8_} cavity (*e.g.* Co^2+^, Ni^2+^, Cu^2+^, Zn^2+^, Mn^2+^, Sc^3+^, Fe^3+^, Lu^3+^) and also guest ions that have not yet been incorporated in the **Pd_12_** cage experimentally, such as Li^+^, Be^2+^, Mg^2+^, Ga^3+^, In^3+^, Sn^4+^, Zr^4+^, and Hf^4+^. In particular, Be^2+^, Fe^3+^, Ga^3+^, and Sn^4+^ seem too small to be hosted efficiently, and consequently a large contraction occurs to maximize the M–O_c_ interactions. When the ion size is between 1.13 and 1.26 Å, a small expansion with Δ*d* less than 0.1 Å is needed, except for Th^4+^. However, a significant distortion of {O_8_} was detected for ions larger than 1.28 Å, such as Ce^3+^, La^3+^ Sr^2+^, Ag^+^, K^+^, Rb^+^, Cs^+^, Ba^2+^, and Ra^2+^, where for some cases elongations larger than 0.4 Å were observed.

It is remarkable that for most of the experimentally observed palladate nanocubes, the distortion induced by guest metal ion encapsulation is no larger than 0.1 Å, and therefore the size matching between the cation guests and the cavity of the **Pd_12_** cage is an important factor that must be considered. Fig. 2 also suggests that guest cations with a size ranging from 0.97–1.26 Å fit well within the {O_8_} cavity in **Pd_12_** regardless of the charge (except Th^4+^). In contrast, smaller (*r* ≤ 0.95 Å) and larger (*r* ≥ 1.28 Å) cations are probably poor candidates for constructing {MPd_12_L_8_} palladate nanocubes from a size-matching point of view, with the optimal cation size being in the 0.97–1.26 Å range.

**Fig. 3:**
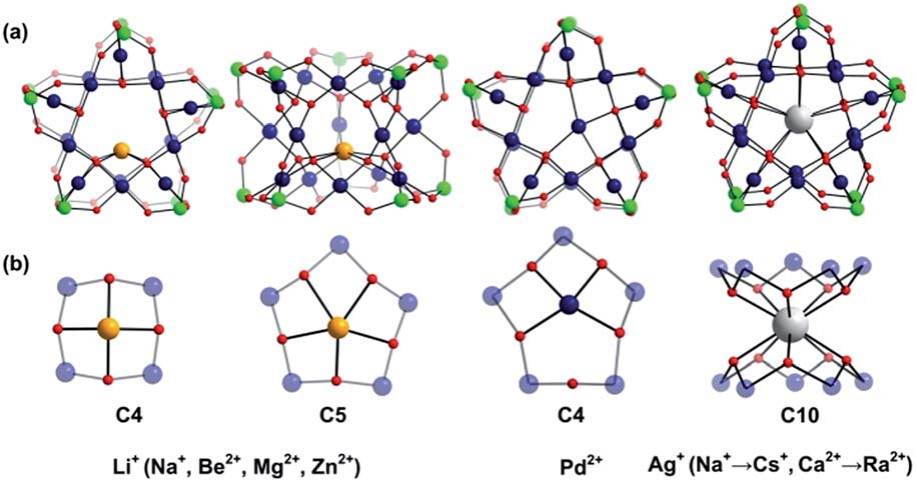
(a) Ball-and-stick representation of the {MPd_15_(AsPh)_10_} palladate cage encapsulated with different guest cations M. The phenyl groups are omitted for clarity; (b) Exclusive view on the central metal ion guest and its coordination sphere. Colour code: guest metal M (orange/dark blue/white), As (green), Pd (dark blue), O (red).

Large guest cations are more likely to accommodate themselves in larger polyoxopalladate assemblies with larger cavities. In order to compare with our computational results on the **Pd_12_** nanocube, we inserted some selected cations (Li^+^, Na^+^, K^+^, Rb^+^, Cs^+^, Ag^+^, Be^2+^, Mg^2+^, Ca^2+^, Sr^2+^, Ba^2+^, Zn^2+^, Pd^2+^) also in the **Pd_15_** nanostar cage. The **Pd_15_** host provides a pentagonal-prismatic {O_10_} inner coordination sphere, which appears too large for encapsulation of small guest cations, such as Li^+^, Be^2+^, Mg^2+^, and Zn^2+^ (*r* < 1.2 Å). In this case the cations move away from the C_5_ symmetry axis of the **Pd_15_** cage and coordinate to less than 10 oxo-ligands. Two types of such off-center coordination modes were observed from our DFT calculations, C_4_ and C_5_ as depicted in Fig. 3. For example, Li^+^ could coordinate to five O_c_ from a Pd_5_O_5_ fragment (2.107, 2.276, 2.261, 2.457, 2.336 Å) or to four O_c_ from a Pd_4_O_4_ fragment (2.002, 2.023, 2.005, 2.022 Å). Although the former configuration includes five coordinated oxygen atoms, the shorter bonds in the latter situation indicate stronger interactions. As expected, the energy differences illustrate that the C_4_ mode is more stable than C_5_ by 6.1 kcal mol^–1^ (Table S2‡). On the other hand, encapsulation of Na^+^, K^+^, Rb^+^, Cs^+^, Ag^+^ Ca^2+^, Sr^2+^, Ba^2+^, and Ra^2+^ (*r* > 1.2 Å) is expected to work well for the **Pd_15_** nanostar cage. Some of them are strongly supported by experiments, which show that **NaPd_15_**, **KPd_15_**, **BaPd_15_**, and **AgPd_15_** can be easily observed in the presence of Na^+^, K^+^, Ba^2+^, and Ag^+^.^10^ It is worth mentioning that Na^+^ prefers to coordinate to a Pd_5_O_5_ face (C_5_) rather than sitting at the body centre of **Pd_15_**, as suggested by experiment.^10*a*^ Interestingly, Pd^2+^ prefers a C_4_ mode, binding to only four O_c_ of the Pd_5_O_5_ face, as shown by XRD.^10a,c^ The good reproducibility of the experimental results by DFT reemphasizes that the size of the cation guest indeed plays an important role in the formation mechanism of the resulting palladate structure.

### 3.2 Complexation energy and competition between Pd^2+^ and other metal cations

**Fig. 4:**
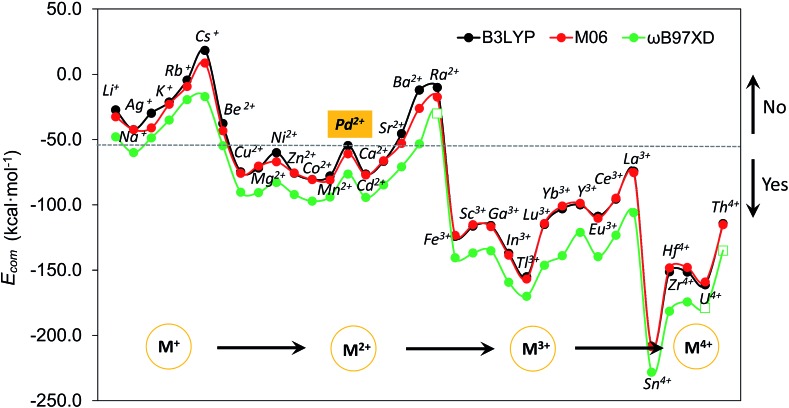
Computed complexation energies (in kcal mol^–1^) of M^*n*+^ (*n* = 1–4) encapsulated in the {Pd_12_(AsPh)_8_} nanocube cage as a function of the cation guest from monovalent to tetravalent at B3LYP (black), M06 (red), and ωB97XD (green) functional level, respectively. The cations are organized by considering both their charge and size. Note that the more negative values indicate a higher affinity of **Pd_12_** for the respective metal ion. The *E*_com_ for Ra, U, and Th at ωB97XD level is not included (square) due to the unavailable van der Waals radius for these elements.

Although size-matching has been established as an essential factor during palladate formation, we have to be aware that (i) the cation–cavity interaction and (ii) the dehydration ability of the cation is not included in the analysis. Thus, we next analyse why the reported cations can be encapsulated by the **Pd_12/15_** cages and predict some potential candidates from an energetic point of view. To rationalize this point, the complexation energy (*E*_com_) of **Pd_12_** and **Pd_15_** for the different guest cations was computed as described in the Computational section, and the results at B3LYP level (black points) are shown in Fig. 4, as well as the energy corrections with M06 (red points) and ωB97XD (green points) functionals. The results for M06 give virtually identical values as those of B3LYP functional, whereas adding the dispersion correction in ωB97XD increases the values of the complexation energies, without changing the overall trends. Therefore, the results at B3LYP level can reasonably identify the trend analysis. Generally, the *E*_com_ becomes more negative (exothermic) as the formal charge of the guest cation increases from +1 to +4. For alkali and alkaline earth ions the *E*_com_ is affected significantly by the size of the cations. All encapsulations seem to be thermodynamically favourable, except for Cs^+^, which shows a positive *E*_com_ of 18.3 kcal mol^–1^. It is illustrative that the first reported polyoxopalladate was **Pd_13_** with a Pd^2+^ ion located at the center,^6^ and that the synthesis occurred in the presence of both Na^+^ and Pd^2+^ ions. Encapsulation of Pd^2+^ in the {Pd_12_(AsPh)_8_} nanocube cage is predicted to be very exothermic with a complexation energy of –54.6 kcal mol^–1^, whereas Na^+^ is much less competitive with –42.2 kcal mol^–1^. Thus, it is not surprising that the Na^+^ ions are not incorporated in the palladate structure, but rather act just as counter cations.

Following such strategy, all cations were divided into two domains with *E*_com_ of Pd^2+^ as a reference (dashed grey line). The *E*_com_ values below the reference line indicate that from a thermodynamic point of view, the respective ions are more favourable to stabilize the **Pd_12_** host cage than the reference ion Pd^2+^. Thus, polyanion nanocubes of the type {MPd_12_(AsPh)_8_} are preferentially formed as compared to {Pd_13_(AsPh)_8_} (**Pd_13_AsPh**). For instance, encapsulation of Fe^3+^ and Sc^3+^ ions inside the **Pd_12_** shell has associated complexation energies of –124.1 ({FePd_12_(AsPh)_8_}) and –116.2 kcal mol^–1^ ({ScPd_12_(AsPh)_8_}), respectively, both being significantly more exothermic than Pd^2+^ (–54.6 kcal mol^–1^, **Pd_13_AsPh**). Indeed, the nanocube family {MPd_12_(AsPh)_8_} with M = Ca^2+^, Co^2+^, Cu^2+^, Ni^2+^, Zn^2+^, Mn^2+^, Sc^3+^, Fe^3+^, Y^3+^, Yb^3+^, Lu^3+^, and Eu^3+^ has already been synthesized by using similar synthetic procedures.^9^ In contrast, encapsulation of M = alkali metal ions, Ag^+^, Be^2+^, Sr^2+^, Ba^2+^, and Ra^2+^ in {MPd_12_(AsPh)_8_} is expected to be difficult due to the less favourable complexation energy with respect to Pd^2+^, in spite of some of them (Li^+^) having a suitable size. In fact, these hypothetical polyoxopalladates have not been synthesized yet in the laboratory. Such conclusions are also valid for nanocube derivatives with other capping groups, such as arsenate (AsO_4_^3–^) and selenite (SeO_3_^2–^), see Fig. S3.‡ The absolute values of *E*_com_ seem to be influenced by the charge of the capping group. Interestingly, almost identical complexation energies were obtained when replacing PhAsO_3_^2–^ by SeO_3_^2–^, which have the same charge and the As^V^–O and Se^IV^–O distances are similar. As based on size only, Ag^+^, K^+^, Rb^+^, Cs^+^, and Ba^2+^ can be excluded as guests for the nanocube cage **Pd_12_**.

We also decided to consider computationally the encapsulation of large guest cations by the 15-palladate nanostar cage {MPd_15_(AsPh)_10_} (Fig. S4‡), and then compare to **Pd_12_**. As expected, the larger guest ions Na^+^, Ag^+^, K^+^, Rb^+^, Ba^2+^, and Ra^2+^ ions were calculated to be both geometrically and energetically suitable for the **Pd_15_** nanostar rather than the **Pd_12_** nanocube, and these results are in full agreement with the experimental facts.^10^ Most of the smaller cations such as Mg^2+^ and Zn^2+^ do not fit geometrically and are also energetically unfavourable in **Pd_15_**. On the other hand, Pd^2+^ shows a similar ability to be encapsulated by the **Pd_12_** nanocube and the **Pd_15_** nanostar, which is consistent with the experimentally observed nanocube **Pd_13_AsPh**,^8^ as well as the mono- and di-palladium-centered nanostar derivatives {Pd ⊂ Pd_15_(PhAsO_3_)_10_} and {Pd_2_ ⊂ Pd_15_(PhAsO_3_)_10_}.^10a,c^ The medium-sized Sr^2+^ ion was shown experimentally to form an unexpected ‘open-nanocube’ structure [SrPd_12_O_6_(OH)_3_(PhAsO_3_)_6_(OAc)_3_]^4–^ (**SrPd_12_-open**),^10*e*^ which inspired us to compute the complexation energies for both the open and closed nanocubes **Pd_12_**, see Table 1. The energy required for encapsulating Sr^2+^ into the open form **SrPd_12_-open** was computed to be –50.0 kcal mol^–1^, and for the closed form [SrPd_12_O_8_(PhAsO_3_)_8_]^6–^ (**SrPd_12_-closed**) it was slightly less exothermic, –45.4 kcal mol^–1^. Therefore, computationally it is predicted that the open form **SrPd_12_-open** is preferentially formed compared to **SrPd_12_-closed**. Amongst the unfavourable guest ions for the closed nanocube shell **Pd_12_-closed**, Sr^2+^ shows the smallest *E*_com_ difference compared to Pd^2+^ with only 9.2 kcal mol^–1^ (and only 4.6 kcal mol^–1^ for **Pd_12_-open**) at the **B3LYP** level, and even smaller differences at the M06 and ωB97XD levels. Thus, a competition between Pd^2+^ and Sr^2+^ guest ions is predicated computationally for such reactions. Experiments showed that only 2% of **SrPd_12_-closed** is formed, and that the Sr^2+^ ion can be substituted by Pd^2+^ to form **Pd_13_AsPh** by simply increasing the pH of the solution.^10*e*^ On the other hand, **SrPd_12_-open** can indeed be isolated in clean form, but to date the **Pd_13_**-open structural type has not been prepared yet.

**Table 1:** Computed complexation energy (kcal mol^–1^) for La^3+^ and Sr^2+^ encapsulated in open and closed **Pd_12_** nanocubes. The experimental product yields are also listed

		B3LYP	M06	ωB97XD	Exp. ratio%
Pd^2+^	Closed	–54.6	–60.8	–76.4	100%
Sr^2+^	Open	–49.9	–61.0	–75.8	98%
	Closed	–45.4	–52.9	–70.9	2%
La^3+^	Open	–70.6	–74.7	–105.7	40%
	Closed	–74.2	–75.2	–105.9	60%

It is interesting to note that *E*_com_ for La^3+^ is very close to that calculated for Pd^2+^, which may lead to mixed products **LaPd_12_-closed** and **Pd_13_AsPh**. In contrast to Sr^2+^, calculations suggest that for La^3+^ the closed nanocube (**LaPd_12_-closed**) is slightly more favourable than the open one (**LaPd_12_-open**), see Table 1. Accordingly, the nature and size of the guest cation directly influences the shape of the resulting poly-oxopalladate and this in turn strongly suggests a template effect of the cation in polypalladate synthesis.

### 3.3 Potential new candidates for the 12-palladate family

The systematic study of the complexation energies for different cation guests has revealed that competition with Pd^2+^ plays a critical role when trying to determine the structure-type of the respective product. Although the energy trend should not be taken quantitatively, it appears to be qualitatively useful in the design of synthetic parameters for discovering new polyoxopalladates.

To date there is a dominance of 3d transition metal and lanthanide elements as central guests of the polyoxo-12-palladate nanocube family {MPd_12_L_8_}. Much less attention has been paid on p-block elements. We calculated the encapsulation of Ga^3+^, In^3+^, and Tl^3+^ as very exothermic with energies of –115.8, –137.1 and –155.1 kcal mol^–1^, respectively. For all three cations, the encapsulation is predicted to be more favourable than for the already reported Sc^3+^ derivative [ScPd_12_O_8_(PhAsO_3_)_8_]^5–^.^9*b*^ In addition, there is a clear trend of increasing encapsulation ability going down group IIIA (B, Al, Ga, In, Tl).

On the other hand, to date no cation with a charge larger than 3+ has been encapsulated in the **Pd_12_** nanocube shell. Therefore, we analysed computationally the encapsulation of several tetravalent cations, such as Sn^4+^, Zr^4+^, Th^4+^, U^4+^, and Hf^4+^. As expected, all tetravalent cations exhibit much favourable complexation energies due to the large anion–cation electrostatic interactions. The small Sn^4+^ ion has the lowest energy of all computed tetravalent ions and is hence the most promising candidate for encapsulation.

In order to verify the various theoretical predictions experimentally, we designed several key experiments, which concern mainly (i) encapsulation of p-block elements in the **Pd_12_** nanocube shell, (ii) synthesis of open- and closed-nanocube isomers for La^3+^, and (iii) competition of three guest cations for **Pd_12_** nanocube shell.

### 3.4 Synthesis and structural characterization of nanocubes **LaPd_12_-closed**, **GaPd_12_**, **InPd_12_** and open-nanocube **LaPd_12_-open**

**Fig. 5:**
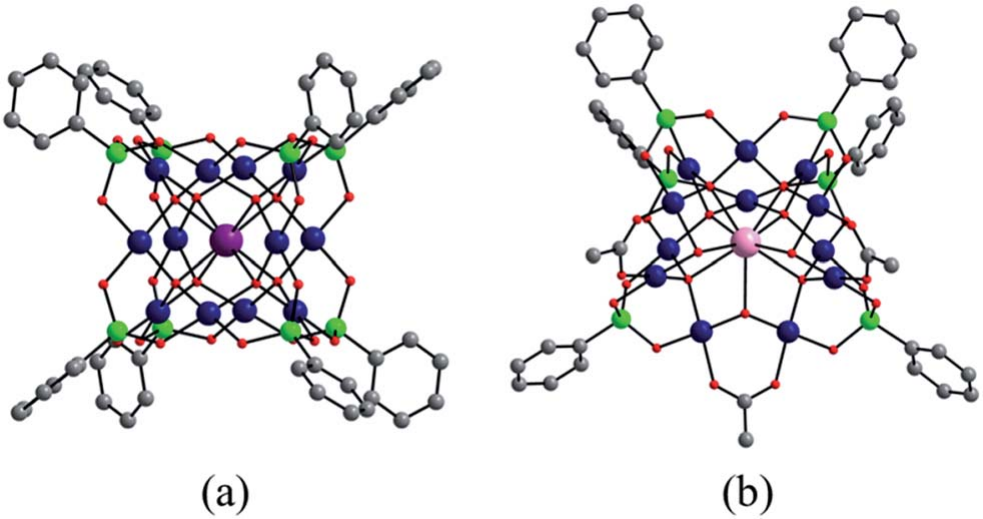
Ball-and-stick representation of (a) the cuboid-shaped {MPd_12_(AsPh)_8_} (M = La^3+^, Ga^3+^, In^3+^) and (b) the open-nanocube **LaPd_12_-open**. Colour code: M (violet), La (pink), Pd (blue), As (green), O (red), C (grey). Hydrogen atoms omitted for clarity.

We have synthesized three new members of the {MPd_12_L_8_} nanocube family, the lanthanum-derivative [LaPd_12_O_8_(PhAsO_3_)_8_]^5–^ (**LaPd_12_-closed**), as well as the first examples of main group III derivatives, namely the gallium-containing [GaPd_12_O_8_(PhAsO_3_)_8_]^5–^ (**GaPd_12_**) and the indium-containing [InPd_12_O_8_(PhAsO_3_)_8_]^5–^ (**InPd_12_**), see Exp. section for details. Single-crystal X-ray analysis revealed that all three polyoxopalladates are isostructural (Fig. 5a, Tables S3 and S4‡). The main differences between **LaPd_12_-closed**, **GaPd_12_**, and **InPd_12_** are the central M–O bond distances with La–O = 2.459(5) Å, Ga–O = 2.211(4) Å, and In–O = 2.293(4) Å, respectively. Notably, La^3+^ is the largest trivalent cation ever encapsulated inside a **Pd_12_** nanocuboid cage and together with the relatively low complexation energy (Table S4‡). **LaPd_12_-closed** presents a good test case for the present study. Interestingly, we are also able to synthesize the open nanocube [LaPd_12_O_6_(OH)_3_(PhAsO_3_)_6_(OAc)_3_]^3–^ (**LaPd_12_-open**, see Fig. 5b). This structure had so far only been observed for strontium(ii) in the center, [SrPd_12_O_6_(OH)_3_(PhAsO_3_)_6_(OAc)_3_]^4–^.^10*e*^ This result perfectly supports the above mentioned calculations (Fig. 4 and Table 1), as the energetically lower closed structure **LaPd_12_-closed** is indeed isolated in higher yields compared to the open structure **LaPd_12_-open**. However, our efforts to prepare additional analogues of **MPd_12_-open** (M = La^3+^, Sr^2+^) with other large lanthanide ions have not been successful, suggesting that La^3+^ is a unique template amongst all lanthanide ions.

The ^13^C and ^1^H NMR spectra indicate good aqueous stability of all four polyanions (Fig. S5 and S6‡). Moreover, we also performed ^71^Ga and ^115^In NMR studies on solutions of **Na-GaPd_12_** and **Na-InPd_12_**, respectively. The observed singlets in ^71^Ga NMR centred at 48.9 ppm (**GaPd_12_**, Fig. S7‡) and the singlet in ^115^In NMR centred at 247.7 ppm (**InPd_12_**, Fig. S8‡) are in full agreement with the solid-state structures. The spectra are clean, indicating that no impurities are present, and the signals are rather narrow, in spite of the quadrupolar nature of both isotopes (^71^Ga, *S* = 3/2; ^115^In, *S* = 9/2), which is a result of the cubic coordination environment around the metal ions combined with the highly symmetrical (cuboctahedral) structure of the overall polyanion, rendering the electric field gradient virtually zero.

We also performed ESI-MS studies in order to study the solution and gas phase properties of **GaPd_12_** and **InPd_12_**. All peaks shown in the spectra can be assigned to species related to **GaPd_12_** and **InPd_12_**, with different numbers of protons or sodium ions attached. For instance, the major envelopes centred at *m*/*z* = 1025.45 (Fig. S9a‡) and *m*/*z* = 1041.45 (Fig. S9b‡) can be attributed to the triply negatively charged [H_2_ **GaPd_12_**]^3–^ and [H_2_ **InPd_12_**]^3–^. Additional MS assignments are summarized in Table S5.‡

### 3.5 Selective encapsulation of Ga^3+^ or In^3+^ in {MPd_12_(AsPh)_8_} nanocube

**Fig. 6:**
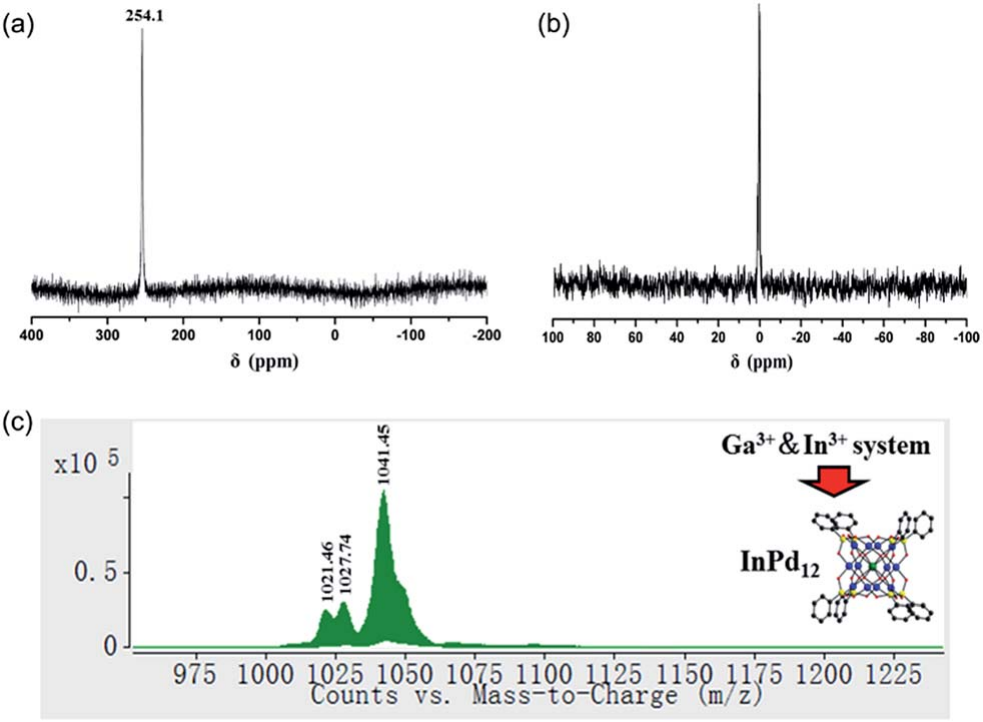
(a) ^115^In NMR spectrum (H_2_O, room temperature, pH = 5.5) of a reaction solution for {MPd_12_(AsPh)_8_} with Ga^3+^ and In^3+^ ions being present in equimolar amounts; (b) ^71^Ga NMR spectrum (H_2_O, room temperature, pH = 5.5) of the same reaction solution after being acidified to pH 1; (c) negative ion ESI mass spectrum of {MPd_12_(AsPh)_8_} crystals collected from the Ga^3+^ and In^3+^ systems in aqueous solution.

To date around 60–70 polyoxopalladates are known, and by far most of them belong to the {MPd_12_L_8_} class of nanocubes with being usually d or f block metal ions.^9^ However, a competitive study using two or more potential guest cations has never been reported before. Considering that both **GaPd_12_** and **InPd_12_** can be followed by NMR in solution, we decided to perform competition studies for this system on fresh reaction solutions. Interestingly, we observed that if equimolar amounts of Ga^3+^ and In^3+^ ions were present, then only **InPd_12_** was formed, as confirmed by ^71^Ga (no signal) and ^115^In NMR (singlet at 254.1 ppm, Fig. 6a). This result indicates that a strong preference exists for **InPd_12_** compared to **GaPd_12_**. The free Ga^3+^ ions were detectable by ^71^Ga NMR at 0 ppm after the solution had been acidified to pH 1 by 1 M HNO_3_ (Fig. 6b).

For the same mixed, equimolar Ga/In system, the filtrated mother solution was allowed to evaporate until the maximum amount of crystals had formed, which were isolated when still covered by mother liquor. These crystals were analysed by ESI-MS and the spectrum obtained showed peaks corresponding exclusively to **InPd_12_**-related species (Fig. 6c). Notably, the envelopes appearing at *m*/*z* = 1021.46 and 1027.74 can be unequivocally ascribed to {Na[InPd_12_O_8_(C_6_H_5_AsO_3_)_7_(AsO_3_)^+^]}^3–^ and {Na[InPd_12_O_8_(C_6_H_5_AsO_3_)_7_(AsO_3_)^+^](H_2_O)}^3–^, which may form from the plenary **InPd_12_** cluster by losing one (C_6_H_5_)^–^ fragment, perhaps during the electrospray ionization processes.

We also performed additional competition experiments, for example for the template pairs Ga^3+^/Sc^3+^ and In^3+^/Sc^3+^, respectively. Both ^71^Ga and ^45^Sc NMR signals could be detected for the Ga^3+^/Sc^3+^ system after the reaction, indicating that **GaPd_12_** and **ScPd_12_** are both formed and coexist in solution (Fig. S10 and S11‡). For the In^3+^/Sc^3+^ system, the ^115^In NMR signal for **InPd_12_** could be detected after a few seconds; whereas the ^45^Sc signal for **ScPd_12_** could only be obtained overnight. These results indicate that selective encapsulation features exist for the central cation guest M of the **Pd_12_** nanocube, which fit well with the trends of the computed complexation energies shown in Fig. 4. Combining the theoretical and experimental results, we obtain a selectivity order of In^3+^ > Ga^3+^ ≈ Sc^3+^. The apparent *E*_com_ difference between In^3+^ (–137.1 kcal mol^–1^) and Ga^3+^/Sc^3+^ (–116.2/–115.8 kcal mol^–1^) leads indeed to a pronounced encapsulation selectivity for In^3+^, whereas Sc^3+^ and Ga^3+^ are more difficult to be separated by polyoxopalladate formation, due to similar complexation energies.

### 3.6 Factors governing encapsulation of metal ion templates in different polyoxopalladate shells

**Fig. 7:**
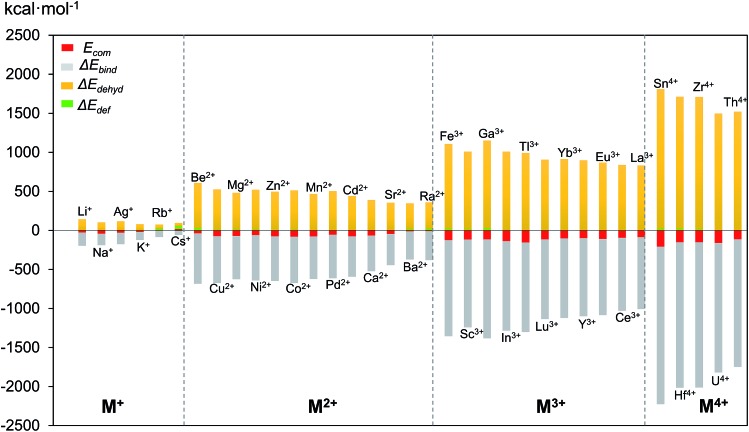
Complexation energy *E*_com_ (in kcal mol^–1^) of M^*n*+^ encapsulated in Pd_12_L_8_ (L = PhAs) host shell and its decomposition terms Δ*E*_dehyd_, Δ*E*_bind_ and Δ*E*_def_.

In the above mentioned theoretical and experimental studies, we have mainly addressed the two main factors that control incorporation of metal ion guests in the polyoxopalladate nanocube shell **Pd_12_**: (i) the energy associated with the complexation of a given cation by the empty **Pd_12_** cage (*E*_com_) and (ii) how the guest fits inside the host cage. In order to gain a deeper understanding of the intrinsic factors governing formation of {MPd_12_L_8_}, we decided to perform an energy decomposition analysis for *E*_com_. From an energetic point of view, we can subdivide the encapsulation process of the cation guest in three steps: (i) dehydration of the cation, (ii) deformation of the **Pd_12_** host shell, and (iii) binding between the cation and the **Pd_12_** host. Thus, *E*_com_ can be expressed as the sum of Δ*E*_def_ + Δ*E*_dehyd_ + Δ*E*_bind_. In Fig. 7, the values computed for Δ*E*_def_, Δ*E*_dehyd_, and Δ*E*_bind_ for a series of cation guests are classified according to the cation charge. It becomes apparent that in absolute values Δ*E*_def_ is much smaller than Δ*E*_dehyd_ and Δ*E*_bind_, and that these two latter terms are very dependent on the cation charge. The complexation energy for di- and trivalent cation guests is respectively two or three times more exothermic than for monovalent cations, due to increasing charge–dipole and charge–charge interactions.

The dehydration energy (Δ*E*_dehyd_) of the cation guest M and the electrostatic interaction (Δ*E*_bind_) between M and the **Pd_12_** nanocage exhibit large values, and in all cases Δ*E*_bind_ is larger than the sum Δ*E*_dehyd_ + Δ*E*_def_, consequently, the *E*_com_ term is always negative and hence exothermic. However, this does not mean that Δ*E*_bind_ alone is sufficient to describe the *E*_com_ trend. In Table 2 three examples are shown indicating that in absolute value Δ*E*_bind_ is indeed dominant, but this term alone does not allow predicting the correct trend for *E*_com_. For example, let us consider the cation guest pair Ga^3+^/In^3+^, for which experiments clearly demonstrated that In^3+^ is captured preferentially over Ga^3+^. The more negative value for *E*_com_ for In^3+^ originates essentially from the large deformation energy of 25.7 kcal mol^–1^ for Ga^3+^ (*vs.* 8.4 kcal mol^–1^ for In^3+^). The other two energy terms (Δ*E*_bind_ and Δ*E*_dehyd_) are significantly larger in absolute terms for Ga^3+^ than In^3+^, but they cancel each other out. Notice that the **Pd_12_** host cage deforms significantly more for Ga^3+^ than In^3+^, because the former is rather small.

**Table 2:** Complexation energy and decomposition analysis terms (all in units of kcal mol^–1^) for three groups of cation guests encapsulated in Pd_12_L_8_ (L = PhAs) host shell

M^*n*+^	*E*_com_	Δ*E*_def_	Δ*E*_bind_	Δ*E*_dehyd_	Ionic radius	Critical energy term
Ga^3+^	–115.8	25.7	–1267.1	1125.5	0.92	Δ*E*_def_
In^3+^	–137.1	8.4	–1146.9	1001.5	1.06	
Lu^3+^	–115.0	5.7	–1021.2	900.5	1.12	Δ*E*_dehyd_
Yb^3+^	–103.0	4.9	–1018.4	910.5	1.13	
Ce^3+^	–95.5	5.8	–935.2	833.9	1.28	Δ*E*_bind_
La^3+^	–86.3	3.9	–920.6	830.4	1.30	

For the Lu^3+^/Yb^3+^ pair, it can be noticed from the values in Table 2 that Lu^3+^ has a more negative complexation energy by 12 kcal mol^–1^. The radii of both ions are virtually identical and the deformation and binding energies are rather similar, and so it can be concluded that the dehydration energy is the critical term in this case.

Finally, the third ion pair Ce^3+^/La^3+^ allows to identify the relevance of electronic structure. The La^3+^ and Ce^3+^ ions have the same charge and essentially identical ionic radii, but the larger atomic number for Ce^3+^ leads to a higher effective nuclear charge and due to the low shielding of f electrons Ce^3+^ has a larger (more negative) binding energy than La^3+^, which in turn leads to a higher (more negative) complexation energy for the former.

In summary, *E*_com_ depends mainly on the following four properties of the metal ion guest: (i) effective ionic radius, (ii) valence state, (iii) dehydration ability, and (iv) electronic configuration and resulting charge-accepting ability. The selectivity for a given cation guest is the result of a delicate balance between the cation–polyoxopalladate and the cation–solvent interactions.

## 4. Conclusions

In host – guest or template-based chemistry, one of the challenges is to know the exact role that ions or small fragments play in the formation of a given species. Ever since the discovery in 2008 of the archetypal polyoxopalladate [H_6_Pd^II^_13_O_8_(As^V^O_4_)_8_]^8–^ (**Pd_13_**), which can be described as a {Pd_12_O_8_(AsO_4_)_8_} nanocube encapsulating a central Pd^2+^ ion with square-planar coordination geometry, about 50 more polyoxopalladate nanocubes {MPd_12_L_8_} with different central metal ion guests M and capping groups L have been reported. However, the main factors that govern the experimental formation of a particular polyoxopalladate (and the non-formation of others) are not well understood. Here, combining experimental and computational chemistry, we have been able to rationalize why a polyoxopalladate shell self-assembles around a particular cation template guest more preferentially than others.

The prototype **Pd_13_** is formed by condensation of [Pd(H_2_O)_4_]^2+^ complex cations in the presence of arsenate anion heterogroups. Nevertheless, if the solution contains other cations M^*n*+^, then in principle {MPd_12_L_8_} type species may also be formed, determined by the favourable complexation energy and the relative competition with respect to Pd^2+^ ions. After an exhaustive computational analysis of complexation and dehydration energies for a series of cation guests we were able to identify the most promising cations to be encapsulated inside the **Pd_12_** nanocube shell. Trivalent and tetravalent cations are easily trapped inside **Pd_12_**, whereas monovalent cations are largely elusive. As based on the calculations, we also performed target-oriented synthetic studies and we were able to isolate four novel polyoxopalladates: (i) the La^3+^-centered nanocube [LaPd_12_O_8_(PhAsO_3_)_8_]^5–^ (**LaPd_12_-closed**), the La^3+^-centered “open” nanocube [LaPd_12_O_6_(OH)_3_(PhAsO_3_)_6_(OAc)_3_]^3–^ (**LaPd_12_-open**), the Ga^3+^-centered [GaPd_12_O_8_(PhAsO_3_)_8_]^5–^ (**GaPd_12_**), and the In^3+^-analogue [GaPd_12_O_8_(PhAsO_3_)_8_]^5–^ (**InPd_12_**). All four compounds were fully characterized in the solid state, in solution, and in the gas phase. In particular ^115^In and ^75^Ga NMR combined with mass spectrometry were very useful, as these techniques allowed to perform speciation studies. We demonstrated that in solutions containing In^3+^ and Ga^3+^ ions only the former is incorporated in the **Pd_12_** shell, due to its more suitable size and higher complexation energy. DFT method also predicted that the large La^3+^ ion should fit in the **Pd_12_** host shell. The experimental work following the computations indeed resulted in the successful synthesis of the regular nanocube **LaPd_12_-closed** as well as the open-shell structure **LaPd_12_-open**. These results reemphasize that size and dehydration energy of the cation guest are the key driving forces in the formation mechanism of nanocuboid polyoxopalladates of the type {MPd_12_L_8_}. Our work has demonstrated how powerful the interplay between theory and experiment can be. We predict that other cations such as Cd^2+^, Tl^3+^, Sn^4+^, Zr^4+^, Hf^4+^, U^4+^, and Th^4+^ amongst others are potential candidates for encapsulation inside the **Pd_12_** host and our efforts are geared in this direction.

## Conflicts of interest

There are no conflicts to declare.
