# Supplementary material for: Size and charge effect of guest cations in the formation of polyoxopalladates: a theoretical and experimental study
Source: Chem Sci. 2017 Sep 25;8(11):7862–72. doi: 10.1039/c7sc03441e (PMC5674179; doi:10.1039/c7sc03441e)
Supplement: Supplementary file 1 [file SC-008-C7SC03441E-s001.pdf]

Electronic Supplementary Material (ESI) for Chemical Science.
This journal is © The Royal Society of Chemistry 2017

Supporting Information for "Size and Charge Effect of Guest Cations in Formation of Polyoxopalladates: A Theoretical and Experimental Study"

Zhongling Lang,[a,‡] Peng Yang,[b,‡] Zhengguo Lin,[b] Likai Yan,[c] Ming-Xing Li,[d] Jorge J. Carbó,[a] Ulrich Kortz,[b,]* and Josep M. Poblet[a,]*

a Departament de Química Física i Inorgànica, Universitat Rovira i Virgili, c/Marcel·lí Domingo 1, 43007 Tarragona, Spain; E-mail: josepmaria.poblet@urv.cat

b Department of Life Sciences and Chemistry, Jacobs University, Campus Ring 1, 28759 Bremen (Germany); E-mail: u.kortz@jacobs-university.de

c Institute of Functional Material Chemistry, Faculty of Chemistry, Northeast Normal University, Changchun 130024, P. R. China

d Department of Chemistry, College of Sciences, Shanghai University, Shanghai 200444, P. R. China

Materials and physical measurements: All reagents were purchased from commercial sources and used without further purification. The NMR spectra of the obtained compounds were recorded on a 400 MHz JEOL ECX instrument at room temperature, using 5-mm tubes for ^{1}H, ^{13}C, ^{71}Ga, ^{115}In, and ^{45}Sc with respective resonance frequencies 399.78 MHz (^{1}H), 100.71 MHz (^{13}C), 122.02 MHz (^{71}Ga), 87.68 MHz (^{115}In), and 97.20 MHz (^{45}Sc). The chemical shifts are reported with respect to the references $Si(CH_3)_4$ (^{1}H and ^{13}C), 0.1 M aqueous $Ga(NO_3)_3$ (^{71}Ga), 0.1 M aqueous $InCl_3$ (^{115}In), and 0.06 M aqueous $Sc(NO_3)_3$. The FT-IR spectra were recorded on KBr disk using a Nicolet-Avatar 370 spectrometer between 400 and 4000 cm^{-1}. Elemental analyses for **Na-LaPd$_{12}$-closed** and **Na-LaPd$_{12}$-open** were performed by CNRS, Service Central d'Analyze, Solaize, France and for **Na-GaPd$_{12}$** and **Na-InPd$_{12}$** by Debrecen University (Debrecen, Hungary). Thermogravimetric analyses (TGA) were carried out on a TA Instruments SDT Q600 thermobalance with a 100 mL min^{-1} flow of nitrogen; the temperature was ramped from 20 °C to 800 °C at a rate of 5 °C min^{-1}. The ESI-MS spectra measurements were made in the negative ion mode on an Agilent 6520 Q-TOF LC/MS mass spectrometer coupled to an Agilent 1200 LC system, and all the MS data were processed by the MassHunter Workstation software. Sample solutions were ca. 10^{-5} M in water and were transferred to the electrospray source by direct injection.

X-ray Crystallography: Crystal data for all compounds were collected at 100 K on a Bruker Kappa X8 APEX CCD single-crystal diffractometer equipped with a sealed Mo tube and a graphite monochromator (λ = 0.71073 Å). The crystals were mounted in a Hampton cryoloop with light oil to prevent loss of crystal waters. The SHELX software package (Bruker) [1] was used to solve and refine the structures. An empirical absorption correction was applied using the SADABS program.[2] The structures were solved by direct methods and refined by the full-matrix least-squares method ($\Sigma w(|F_o|^2 - |F_c|^2)^2$) with anisotropic thermal parameters for all heavy atoms included in the model. The hydrogen atoms of the phenyl rings and acetate ligands were introduced in geometrically calculated positions. The H atoms of the crystal waters were not located. It was not possible to localize all sodium counter cations by XRD, due to crystallographic disorder, which is a common problem in polyoxometalate crystallography. Therefore, the exact number of counter cations and crystal waters in the formulas were based on elemental analysis, and used throughout the manuscript and in the CIF file for overall consistency. The crystal data and structure refinement for all four compounds are summarized in Table S1. CCDC-1555482 (**Na-LaPd$_{12}$-closed**), CCDC-1555483 (**Na-LaPd$_{12}$-open**), CCDC-1555480 (**Na-GaPd$_{12}$**), and CCDC-1555481 (**Na-InPd$_{12}$**) contain the supplementary crystallographic data for this paper. These data can be obtained free of charge from The Cambridge Crystallographic Data Center via www.ccdc.cam.ac.uk/data_request/cif.

Bond valence sum calculations: The bond valence sum (BVS) calculations were performed with a program copyrighted by Chris Hormillosa & Sean Healy and distributed by I. D. Brown.[3]

The calculated BVS values for the structurally inequivalent oxygens in four compounds are presented in Table S2. These values show monoprotonation of the three unique μ_3-bridging oxygens of **Na-LaPd$_{12}$-open** (O3LA, O5LA and O7LA).

FT-IR spectra of Na-LaPd$_{12}$-closed, **Na-LaPd$_{12}$-open, Na-GaPd$_{12}$** and **Na-InPd$_{12}$:** Except for the absence of strong absorption peaks belong to $\nu(CH_3COO)$ in **Na-LaPd$_{12}$-open**, four polyoxopalladate salts exhibit similar FT-IR spectra showing only slight shifts in some band positions (Figure S11 – S14). As an example, for **Na-LaPd$_{12}$-open**, the strong band at 536 cm^{-1} correspond to the different vibrational modes of the Pd–O groups. The strong band at 814 cm^{-1} is designated to the vibrations of the $\{AsO_3\}$ fragments. The stretching and bending vibrations of the C–H and C–C bonds of the phenyl rings occur in the regions between 1485 – 1093 cm^{-1} and 744 – 694 cm^{-1}, respectively. Moreover, characteristic absorption band at 1539 cm^{-1} corresponds to the asymmetric stretching vibration of COO^- groups, whereas the one at 1419 cm^{-1} is assigned to their symmetric vibration. The separation between the $\nu_{as}(COO)$

and ν_s(COO) band is less than 200 cm^{-1}, which confirms the bidentate chelating mode of the carboxylate group.[4] The broad band near 1633 cm^{-1} belongs to asymmetric vibrations of the crystal waters.[5]

TGA of Na-LaPd$_{12}$-closed, **Na-LaPd$_{12}$-open, Na-GaPd$_{12}$** and **Na-InPd$_{12}$:** The thermal stability of four compounds were investigated on crystalline samples by thermogravimetric analysis under a nitrogen atmosphere, and three continuous weight-loss steps were observed on the TGA curves of all four samples, exhibiting similar thermogravimetric processes (Figure S15 – S18).

References

1. G. M. Sheldrick, *Acta Crystallogr., Sect. A: Found. Crystallogr.* 2008, **A64**, 112.
2. G. M. Sheldrick, *SADABS, Program for empirical X-ray absorption correction*; Bruker-Nonius: Madison, WI, 1990.
3. I. D. Brown and D. Altermatt, *Acta Crystallogr.* 1985, **B41**, 244.
4. G. B. Deacon and R. J. Phillips, *Coord. Chem. Rev.* 1980, **33**, 227.
5. K. Nakamoto, *Infrared and Raman Spectra of Inorganic and Coordination Compounds- Part A: Theory and Applications in Inorganic Chemistry, 5th ed.;* Wiley and Sons, New York, **1997**.

Table S1. Comparison of DFT-computed and X-ray distance (Å) for the {$MPd_{12}(AsPh)_8$} complexes with different metal guest ions encapsulated in, and the corresponding effective ionic radius (Å) for each cation in 8-coordinated environment was listed.

M	O_c—O_c	M—O_c	M—Pd	*r*
Li^{+}	2.716/2.726	2.358	3.404	1.06
Na^{+}	2.797/2.805	2.427	3.430	1.32
Ag^{+}	2.886/2.888	2.500	3.473	1.42
K^{+}	3.025	2.619	3.497	1.65
Rb^{+}	3.223/3.234	2.794	3.560	1.75
Cs^{+}	3.423/3.425	2.966	3.622	1.88
Be^{2+}	2.550/2.546	2.206	3.343	~0.80
Cu^{2+}	2.698/2.688 (2.270)	2.486/2.170 (2.631)	3.393 (3.327)	~1.03
Mg^{2+}	2.643/2.647	2.290	3.384	1.03
Ni^{2+}	2.629/2.671(2.608/2.640)	2.289 (2.277)	3.367/3.392 (3.317/3.318)	~1.00
Zn^{2+}	2.687/2.692 (2.630)	2.329 (2.269)	3.399 (3.329)	1.04
Co^{2+}	2.659/2.662 (2.574)	2.303 (2.259)	3.390 (3.320)	1.04
Mn^{2+}	2.711 (2.662)	2.347 (2.294)	3.410 (3.341)	1.10
Pd^{2+}	2.767/2.751 (2.697/2.705)	2.281/2.491 (2.244/2.437)	3.418/3.422 (3.352/3.358)	~1.20
Cd^{2+}	2.830/2.814	2.446	3.447	1.24
Ca^{2+}	2.827/2.836 (2.737/2.760)	2.453(2.384)	3.452(3.372/3.380)	1.26
Sr^{2+}	2.953/2.956	2.559	3.491	1.40
Ba^{2+}	3.149/3.154	2.729	3.543	1.56
Ra^{2+}	3.209/3.215	2.782	3.560	1.62
Fe^{3+}	2.611/2.614 (2.561)	2.262 (2.224)	3.375 (3.309)	0.92
Sc^{3+}	2.627/2.641 (2.590)	2.279 (2.257)	3.388 (3.310)	1.01
Ga^{3+}	2.576/2.569(2.555)	2.227(2.212)	3.357(3.304)	~0.92
In^{3+}	2.679/2.681(2.667/2.685)	2.321(2.315)	3.405(3.344)	1.06
Tl^{3+}	2.753/2.757(2.714)	2.386(2.351)	3.430((3.372/3.384)	1.12
Lu^{3+}	2.712/2.715 (2.654/2.671)	2.350 (2.304)	3.421 (3.358/3.368)	1.12
Yb^{3+}	2.738/2.751(2.689/2.692)	2.363/2.381(2.329)	3.427(3.364)	1.13
Y^{3+}	2.780/2.788 (2.700/2.713)	2.412 (2.346)	3.442 (3.385)	1.16
Eu^{3+}	2.811/2.816 (2.744/2.753)	2.436 (2.381)	3.455 (3.381/3.389)	1.21
Ce^{3+}	2.891/2.908	2.501	3.473/3.482	1.28
La^{3+}	2.957/2.967	2.564	3.503/3.498	1.30
Sn^{4+}	2.574/2.581	2.231	3.373	0.95
Hf^{4+}	2.622/2.636	2.275	3.390/3.396	0.97
Zr^{4+}	2.655/2.660	2.301	3.405	0.98
U^{4+}	2.750/2.780	2.399	3.444	1.14
Th^{4+}	2.923/2.929	2.537	3.491	1.19

Table S2. Relative energies (kcal·mol^{-1}) for cation encapsulated in Pd_{15} at different sites.

	Sites	ΔE		Sites	ΔE
Li^{+}	C_4	0.0	Mg^{2+}	C_4	0.0
	C_5(on Pd_5O_5)	6.1		C_5	3.9
Na^{+}	C_4	1.2	Ca^{2+}	*Center*	
	C_5 (on Pd_5O_5)	0.0	Sr^{2+}	*Center*	
	Center	0.4	Ba^{2+}	*Center*	
K^{+}	*Center*		Ra^{2+}	*Center*	
Rb^{+}	*Center*		Zn^{2+}	C_4	
Cs^{+}	*Center*			C_5	3.2
Ag^{+}	*Center*		Pd^{2+}	C_4 (on Pd_5O_5)	0.0
Be^{2+}	C_3			C_5	8.1
				C_6	4.9

Table S3. Crystal data and structure refinement for **Na-LaPd$_{12}$-closed**, **Na-LaPd$_{12}$-open**, **Na-GaPd$_{12}$**, and **Na-InPd$_{12}$**.

Empirical formula	$Na_5LaPd_{12}As_8$ $C_{48}H_{102}O_{63}$	$Na_3LaPd_{12}As_6$ $C_{42}H_{122}O_{73}$	$Na_5GaPd_{12}As_8$ $C_{48}H_{112}O_{68}$	$Na_5InPd_{12}As_8$ $C_{48}H_{100}O_{62}$
Formula weight, g/mol	3817.31	3729.59	3838.20	3775.20
Crystal system	Tetragonal	Triclinic	Tetragonal	Tetragonal
Space group	*I*4/*m*	*P*$\bar{1}$	*I*4/*m*	*I*4/*m*
a, Å	15.8839(6)	13.0985(9)	15.8084(9)	15.7992(6)
b, Å	15.8839(6)	16.2291(12)	15.8084(9)	15.7992(6)
c, Å	25.1431(19)	22.9839(14)	24.853(3)	24.954(2)
α, °	90	103.266(3)	90	90
β, °	90	92.094(3)	90	90
γ, °	90	97.633(4)	90	90
Volume, Å^3	6343.6(7)	4701.9(6)	6210.9(10)	6228.9(7)
Z	2	1	2	2
D_{calc}, g/cm^3	1.998	1.317	2.052	2.013
Absorption coefficient, mm^{-1}	4.153	2.453	4.125	4.075
F(000)	3644	1798	3692	3608
Theta range for data collection, °	1.52 to 28.32	1.30 to 28.33	1.53 to 28.28	1.53 to 28.32
Completeness to Θ_{max}	99.9 %	99.3 %	99.9 %	99.9 %
Index ranges	-21<=h<=20, -20<=k<=20, -33<=l<=33	-17<=h<=17, -21<=k<=21, -30<=l<=30	-21<=h<=21, -20<=k<=15, -33<=l<=33	-21<=h<=21, -21<=k<=21, -33<=l<=33
Reflections collected	57287	160109	115016	65892
Independent reflections	4052	23219	3949	3971
R(int)	0.0453	0.0608	0.0415	0.0490
Absorption correction	Semi-empirical from equivalents	Semi-empirical from equivalents	Semi-empirical from equivalents	Semi-empirical from equivalents
Data / restraints / parameters	4052 / 27 / 83	23219 / 0 / 497	3949 / 34 / 87	3971 / 0 / 87

Goodness-of-fit on F^2	1.083	1.081	1.036	1.012
R_1,[a] wR_2[b] (I> 2σ(I))	R_1 = 0.0560, wR_2 = 0.1967	R_1 = 0.0845, wR_2 = 0.2376	R_1 = 0.0547, wR_2 = 0.1710	R_1 = 0.0552, wR_2 = 0.1960
R_1,[a] wR_2[b] (all data)	R_1 = 0.0639, wR_2 = 0.2129	R_1 = 0.1092, wR_2 = 0.2626	R_1 = 0.0665, wR_2 = 0.1953	R_1 = 0.0647, wR_2 = 0.2143
Largest diff. peak and hole, e/Å^3	4.882 and -2.688	5.252 and -4.815	2.982 and -2.630	3.139 and -2.612

[a] $R_1 = \Sigma||F_o| - |F_c||/\Sigma|F_o|$. [b] $wR_2 = [\Sigma w(F_o^2 - F_c^2)^2/\Sigma w(F_o^2)^2]^{1/2}$.

Table S4. Bond valence sum values for different structural types of oxygen atoms in **$LaPd_{12}$-closed**, **$LaPd_{12}$-open**, **$GaPd_{12}$**, and **$InPd_{12}$**.

**$LaPd_{12}$-closed**			
**μ_4-O (La, 3Pd)**	**BVS value**	O1A	1.727
O1LA	2.163	O2A	1.729
**μ_2-O (Pd–O–As)**	**BVS value**	O3A	1.751
**$LaPd_{12}$-open**			
**μ_4-O (La, 3Pd)**	**BVS value**	**μ_2-O (Pd–O–As)**	**BVS value**
O1LA	2.142	O2A2	1.735
O2LA	2.094	O3A2	1.769
O4LA	2.066	O1A3	1.718
O6LA	2.085	O2A3	1.784
O8LA	2.072	O3A3	1.815
O9LA	2.098	O1A4	1.765
**μ_3-O (La, 2Pd)**	**BVS value**	O2A4	1.803
O3LA	1.384	O3A4	1.685
O5LA	1.379	O1A5	1.815
O7LA	1.402	O2A5	1.799
**μ_2-O (Pd–O–As)**	**BVS value**	O3A5	1.777
O1A1	1.743	O1A6	1.787
O2A1	1.739	O2A6	1.782
O3A1	1.751	O3A6	1.690
O1A2	1.792		
**$GaPd_{12}$**			
**μ_4-O (Ga, 3Pd)**	**BVS value**	O1A	1.764
O1GA	2.135	O2A	1.743
**μ_2-O (Pd–O–As)**	**BVS value**	O3A	1.750
**$InPd_{12}$**			
**μ_4-O (In, 3Pd)**	**BVS value**	O1A	1.728
O1IN	2.157	O2A	1.763
**μ_2-O (Pd–O–As)**	**BVS value**	O3A	1.754

Table S5. Assignments and *m*/*z* values for the main peaks observed in the ESI-MS spectra of **Na-GaPd$_{12}$** and **Na-InPd$_{12}$**.

m/z	Formula
**1032.78**	$[HNa\mathbf{GaPd_{12}}]^{3-}$
**1040.11**	$[Na_2\mathbf{GaPd_{12}}]^{3-}$
m/z	**Formula**
**1047.78**	$[HNa\mathbf{InPd_{12}}]^{3-}$

Table S6. Complexation energy E_{com} (in kcal·mol^{-1}) of M^{n+} encapsulated in $Pd_{12}L_8$ (L = PhAs) host shell and its decomposition terms ΔE_{dehyd}, ΔE_{bind} and ΔE_{def}.

M^{n+}	E_{co}	ΔE_{deform}	ΔE_{bind}	ΔE_{dehyd}	r
Li^+	–27.2	2.7	–170.9	140.9	1.06
Na^+	–42.2	1.1	–146.7	103.4	1.32
Ag^+	–29.7	1.3	–147.6	116.6	1.42
K^+	–21.2	5.7	–102.0	75.0	1.65
Rb^+	–4.5	20.6	–80.2	55.0	1.75
Cs^+	18.3	35.3	–59.2	42.2	1.88
Be^{2+}	–37.7	28.8	–646.8	580.3	~0.80
Cu^{2+}	–74.8	11.1	–600.4	514.5	~1.03
Mg^{2+}	–71.7	12.3	–553.5	469.5	1.03
Ni^{2+}	–60.0	13.2	–581.7	508.5	~1.00
Zn^{2+}	–75.7	6.2	–572.2	490.2	1.10
Co^{2+}	–80.5	9.9	–594.2	503.8	1.04
Mn^{2+}	–77.9	4.4	–545.2	462.9	1.04
Pd^{2+}	–54.6	4.7	–558.3	499.0	~1.20
Cd^{2+}	–76.4	1.9	–518.0	439.7	1.24
Ca^{2+}	–66.6	1.9	–456.2	387.6	1.26
Sr^{2+}	–45.4	2.8	–400.7	352.5	1.40
Ba^{2+}	–11.9	15.3	–360.2	332.9	1.56
Ra^{2+}	–10.1	19.9	–370.0	340.0	1.62
Fe^{3+}	–124.1	19.9	–1232.3	1088.4	0.92
Sc^{3+}	–116.2	16.0	–1126.1	993.9	1.01
Ga^{3+}	–115.8	25.7	–1267.1	1125.5	~0.92
In^{3+}	–137.1	8.4	–1146.9	1001.5	1.06
Tl^{3+}	–155.1	3.1	–1147.5	989.2	1.12
Lu^{3+}	–115.0	5.7	–1021.2	900.5	1.12

Yb^{3+}	–103.0	4.9	–1018.4	910.5	1.13
Y^{3+}	–99.9	2.6	–999.7	897.1	1.16
Eu^{3+}	–108.7	3.3	–977.5	865.4	1.21
Ce^{3+}	–95.5	5.8	–935.2	833.9	1.28
La^{3+}	–86.3	3.9	–920.6	830.4	1.30
Sn^{4+}	–208.2	20.9	–2018.6	1789.5	0.95
Hf^{4+}	–151.2	20.1	–1864.6	1693.3	0.97
Zr^{4+}	–151.4	14.2	–1861.0	1695.4	0.98
U^{4+}	–161.2	4.7	–1659.3	1493.4	1.14
Th^{4+}	–114.3	3.7	–1635.3	1517.3	1.19

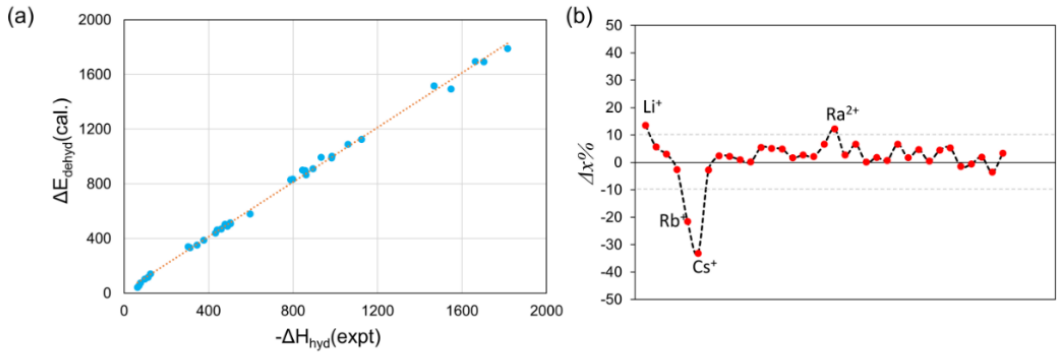

Figure S1. (a) Comparison of experimental hydration enthalpies and computational dehydration energies for all selected guest metal ions; (b) the deviation between experimental and theoretical values $\Delta x\% = \frac{\Delta E_{dehyd} - (-H_{hyd})}{-H_{hyd}}\%$.

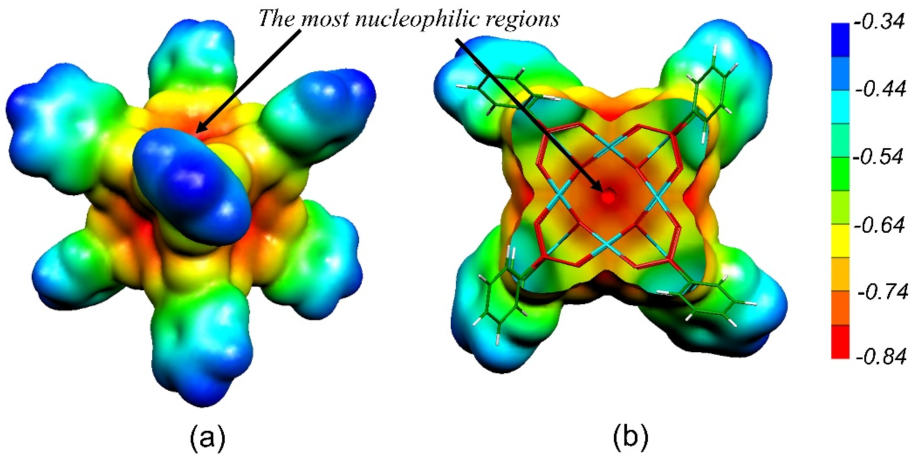

Figure S2. Side (a) and top (b, one half of the molecule) view of the MEP distribution for $[Pd_{12}O_8(PhAsO_3)_8]^{8-}$, the values taken by EP at each point are coded by colors: red-yellow for nucleophilic regions and green-blue for electrophilic regions. The cavity and six Pd_4O_4 interface are the most nucleophilic regions.

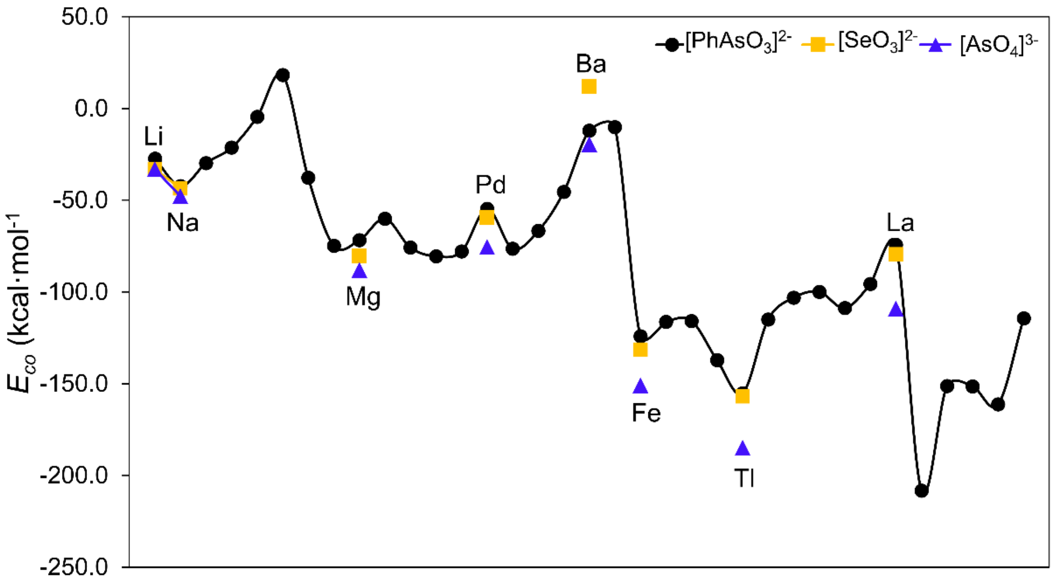

Figure S3. The complexation energies (kcal·mol^{-1}) for cations (Li^+, Na^+, Mg^{2+}, Pd^{2+}, Ba^{2+}, Fe^{3+}, Tl^{3+}, and La^{3+}) encapsulated in $Pd_{12}L_8$ with different capping groups. (L = $PhAs^VO_3^{2-}$, black points; $Se^{IV}O_3^{2-}$, orange square; $As^VO_4^{3-}$, blue triangle).

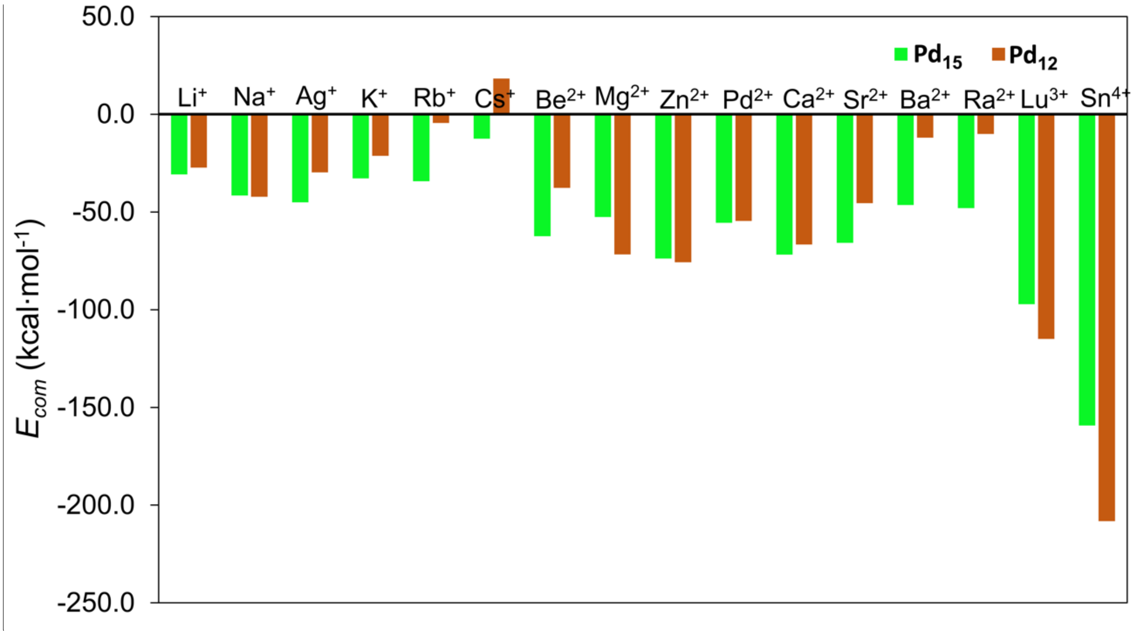

Figure S4. The complexation energies (kcal·mol^{-1}) comparison for selected cations encapsulated in the cage of $\{MPd_{12}(AsPh)_8\}$ and $\{MPd_{15}(AsPh)_{10}\}$ respectively.

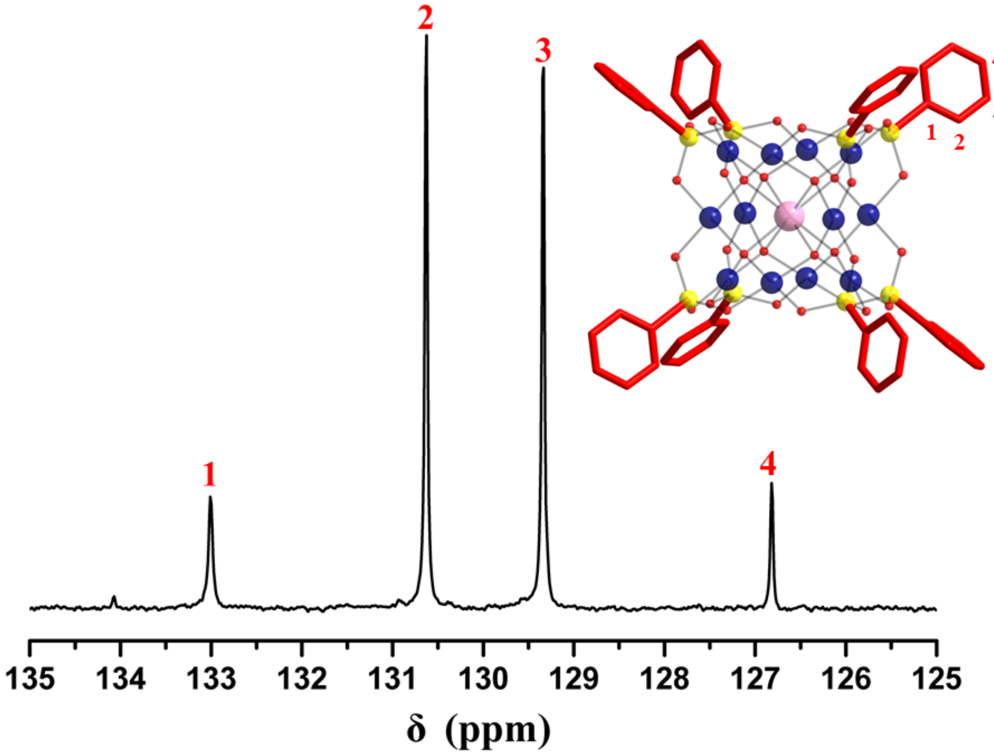

1
2
3
4
135 134 133 132 131 130 129 128 127 126 125
δ (ppm)

(a)

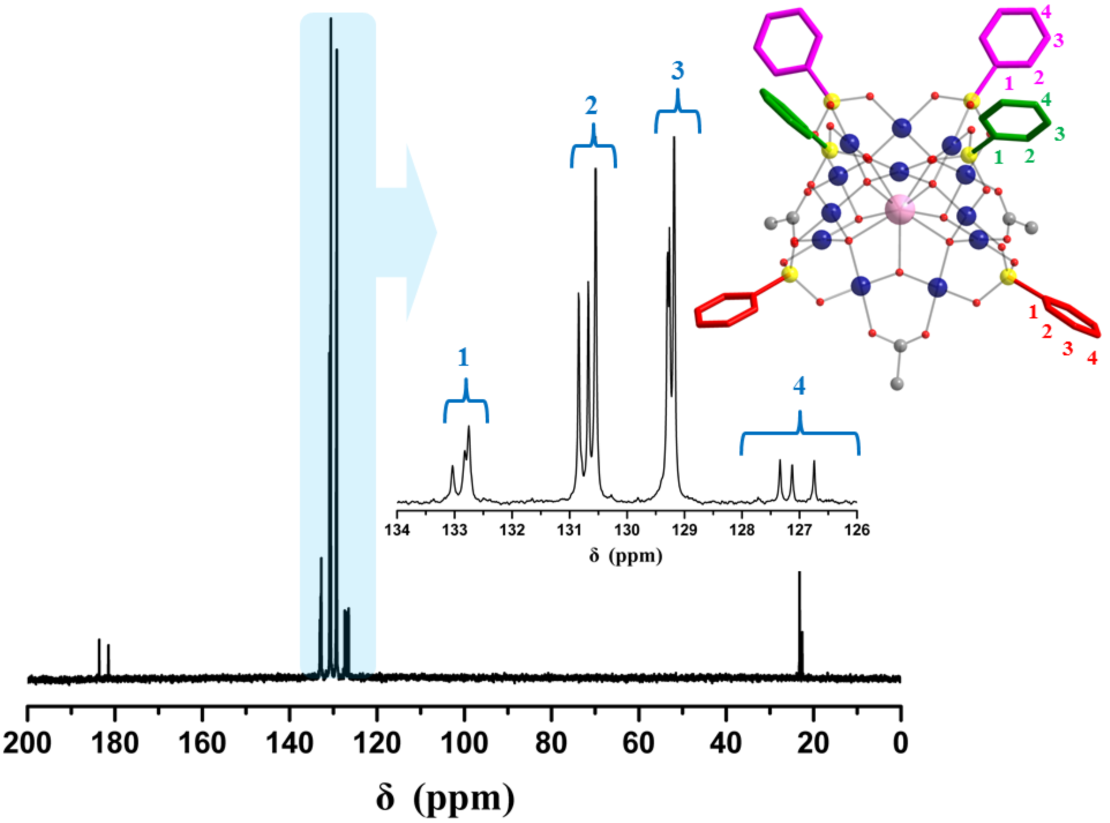

1
2
3
4
134 133 132 131 130 129 128 127 126
δ (ppm)
200 180 160 140 120 100 80 60 40 20 0
δ (ppm)

(b)

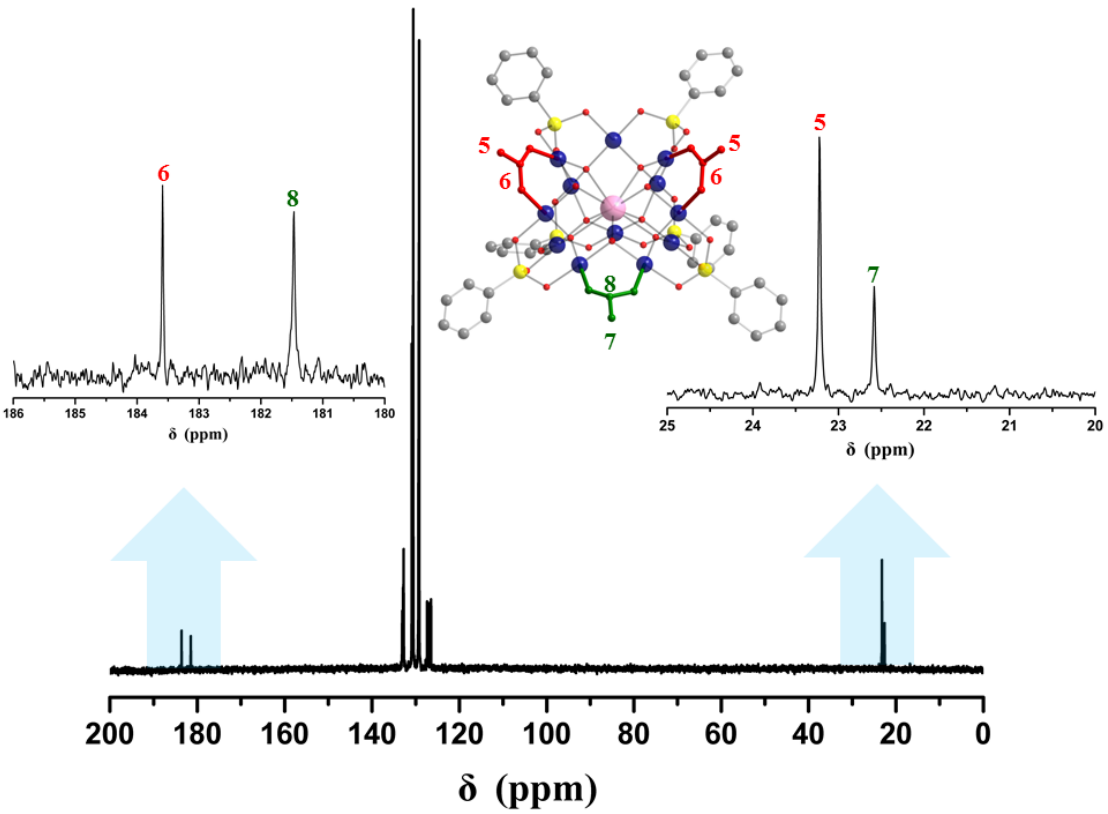
6
8
186
185
184
183
182
181
180
δ (ppm)
5
5
6
6
8
7
5
7
25
24
23
22
21
20
δ (ppm)
200
180
160
140
120
100
80
60
40
20
0
δ (ppm)

(c)

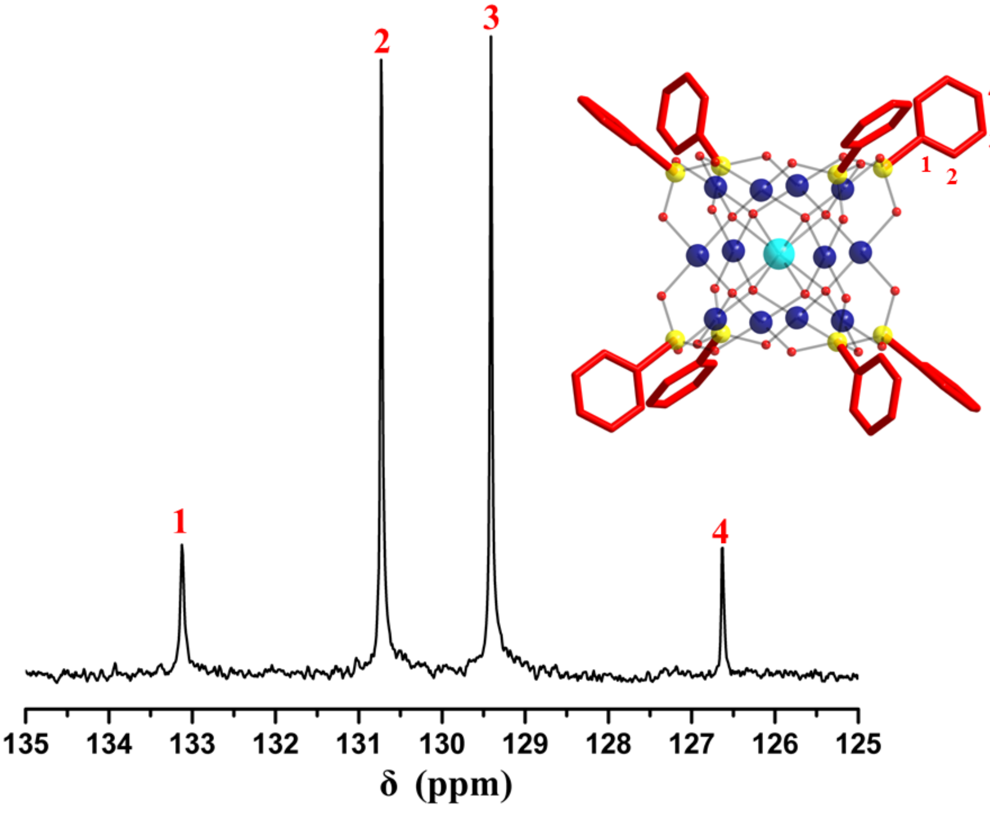
1
2
3
4
4
3
1
2
135
134
133
132
131
130
129
128
127
126
125
δ (ppm)

(d)

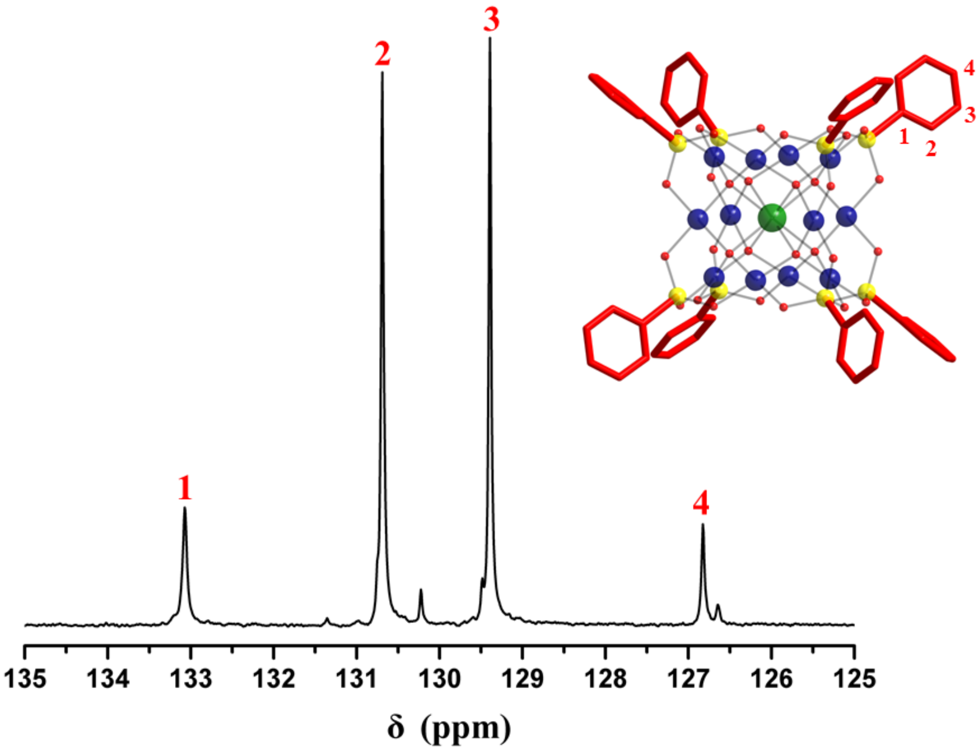

(**e**)

Figure S5. ^{13}C NMR spectra of **Na-LaPd$_{12}$-closed** (**a**), **Na-LaPd$_{12}$-open** (**b** and **c**), **Na-GaPd$_{12}$** (**d**), and **Na-InPd$_{12}$** (**e**) recorded in H_2O/D_2O at room temperature.

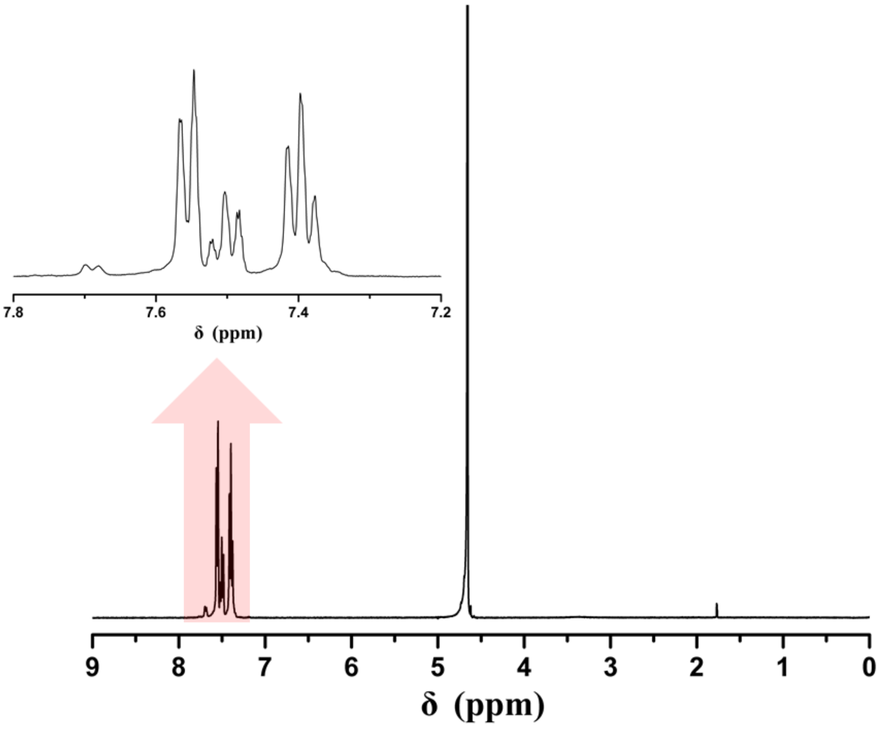

(**a**)

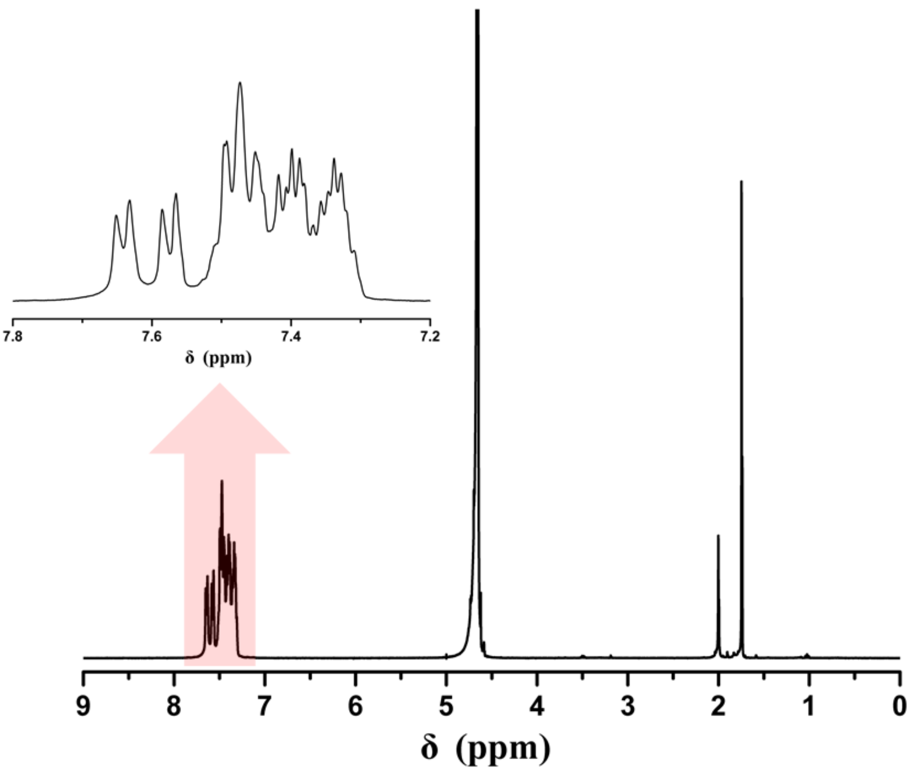
7.8
7.6
7.4
7.2
δ (ppm)
9
8
7
6
5
4
3
2
1
0
δ (ppm)

(b)

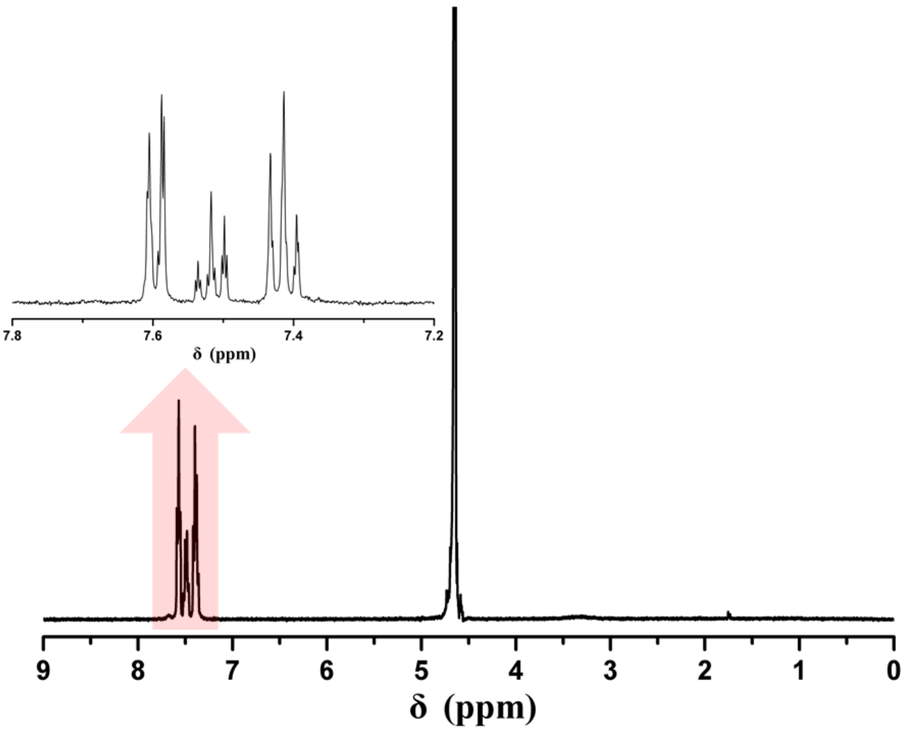
7.8
7.6
7.4
7.2
δ (ppm)
9
8
7
6
5
4
3
2
1
0
δ (ppm)

(c)

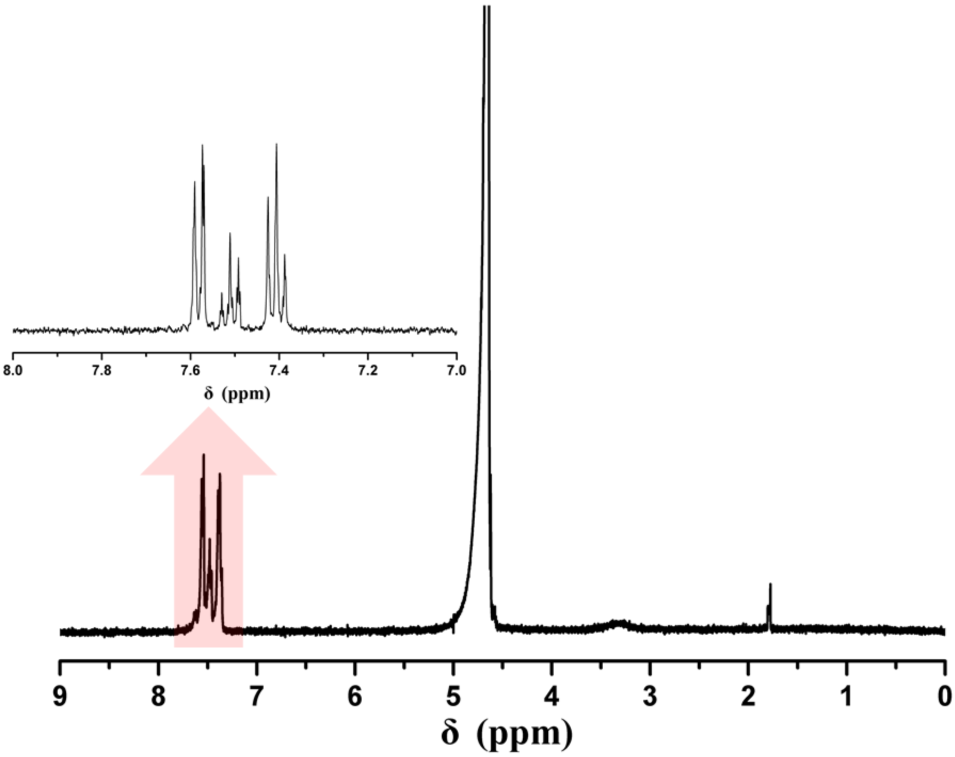

(d)

Figure S6. ^{1}H NMR spectra of **Na-LaPd$_{12}$-closed** (**a**), **Na-LaPd$_{12}$-open** (**b**), **Na-GaPd$_{12}$** (**c**), and **Na-InPd$_{12}$** (**d**) recorded in D_2O at room temperature.

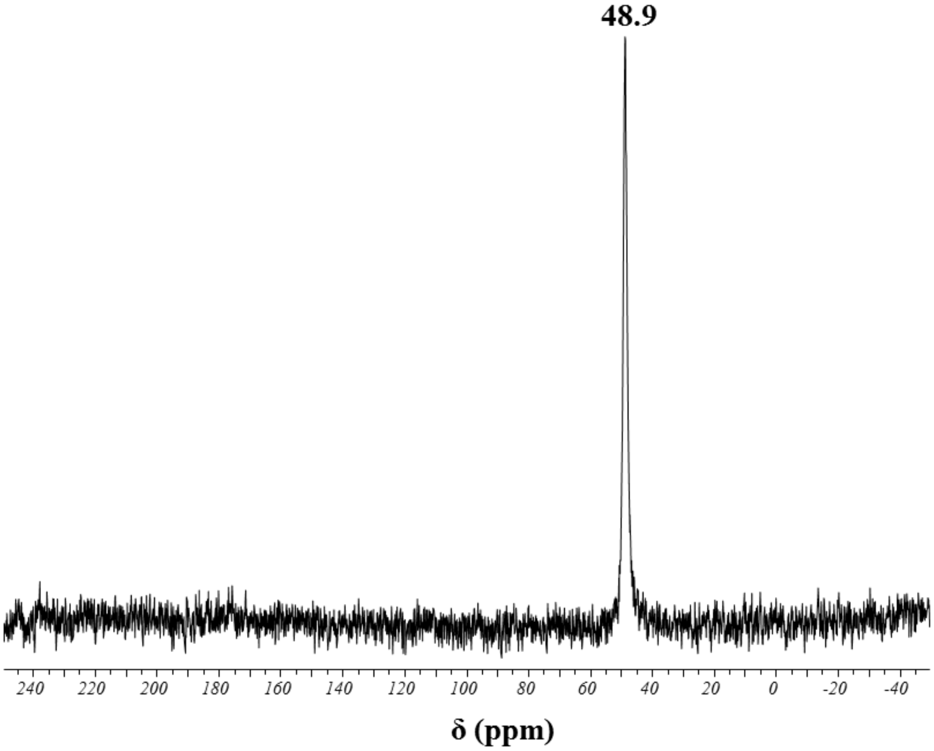

Figure S7. [71]Ga NMR spectrum of **Na-GaPd$_{12}$** recorded in H_2O at room temperature.

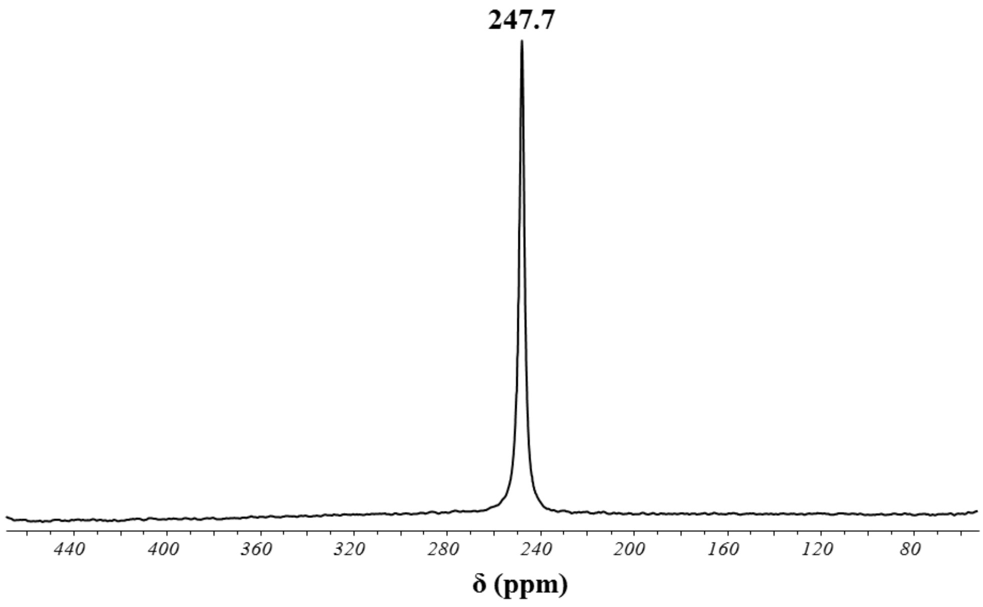

Figure S8. ^{115}In NMR spectrum of **Na-InPd$_{12}$** recorded in H_2O at room temperature.

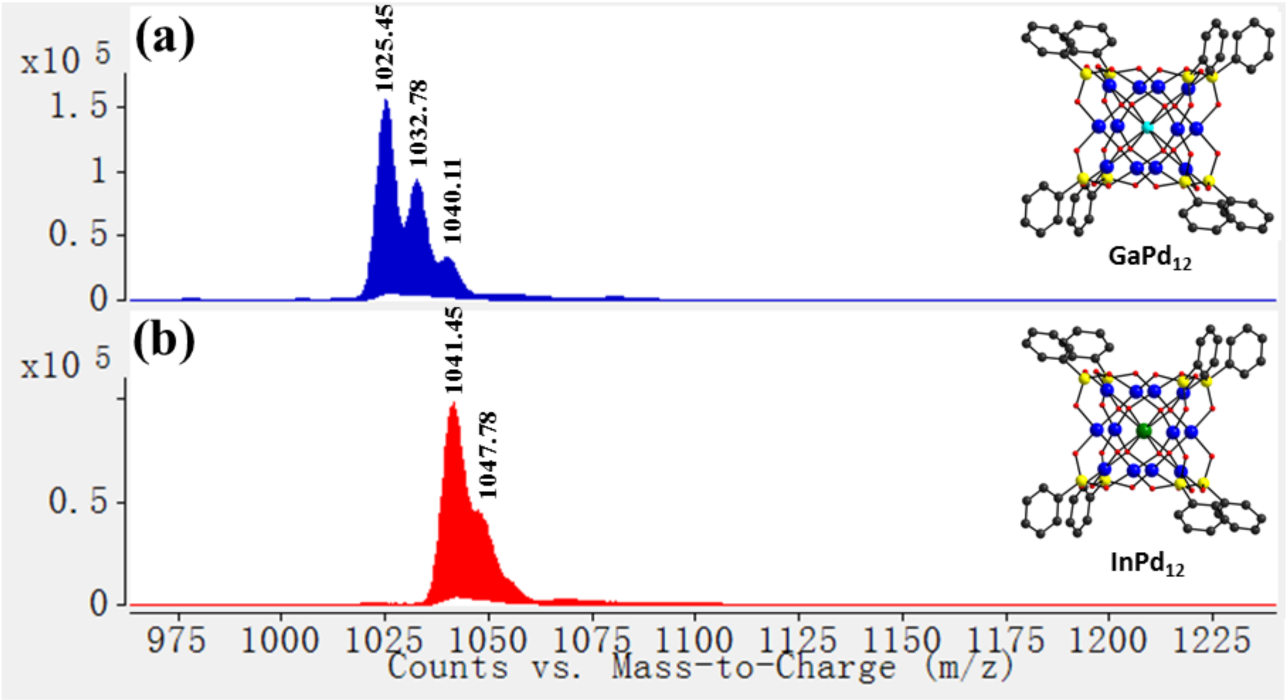

Figure S9. Negative ion mass spectra of **Na-GaPd$_{12}$** (**a**) and **Na-InPd$_{12}$** (**b**) in aqueous solution.

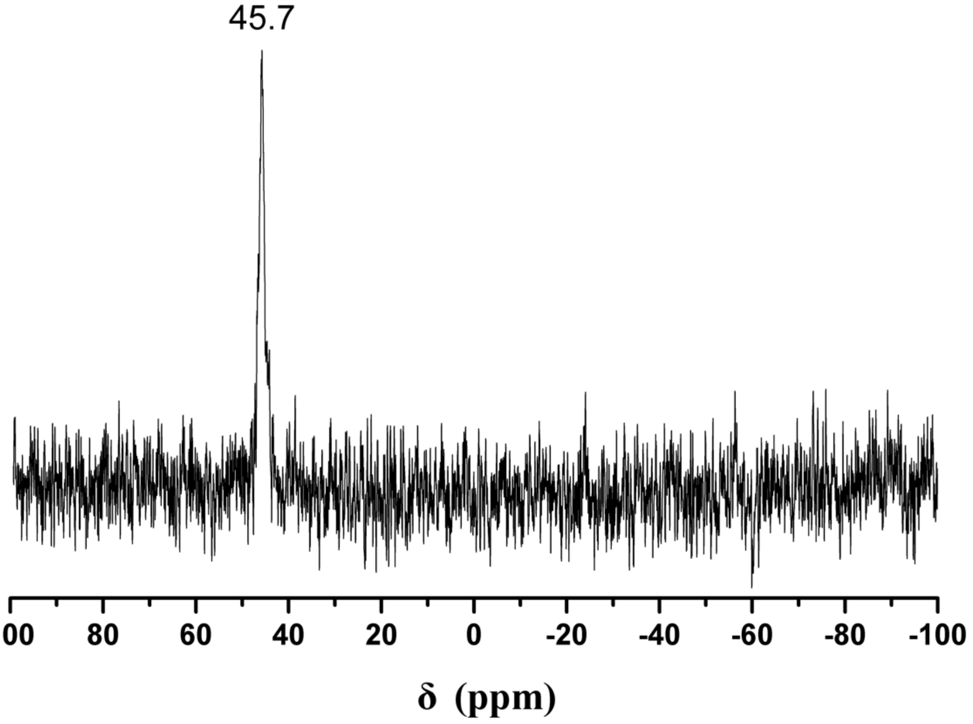

Figure S10. ^{71}Ga NMR spectrum of the reaction solution of Ga^{3+} & Sc^{3+} system recorded in H_2O at room temperature.

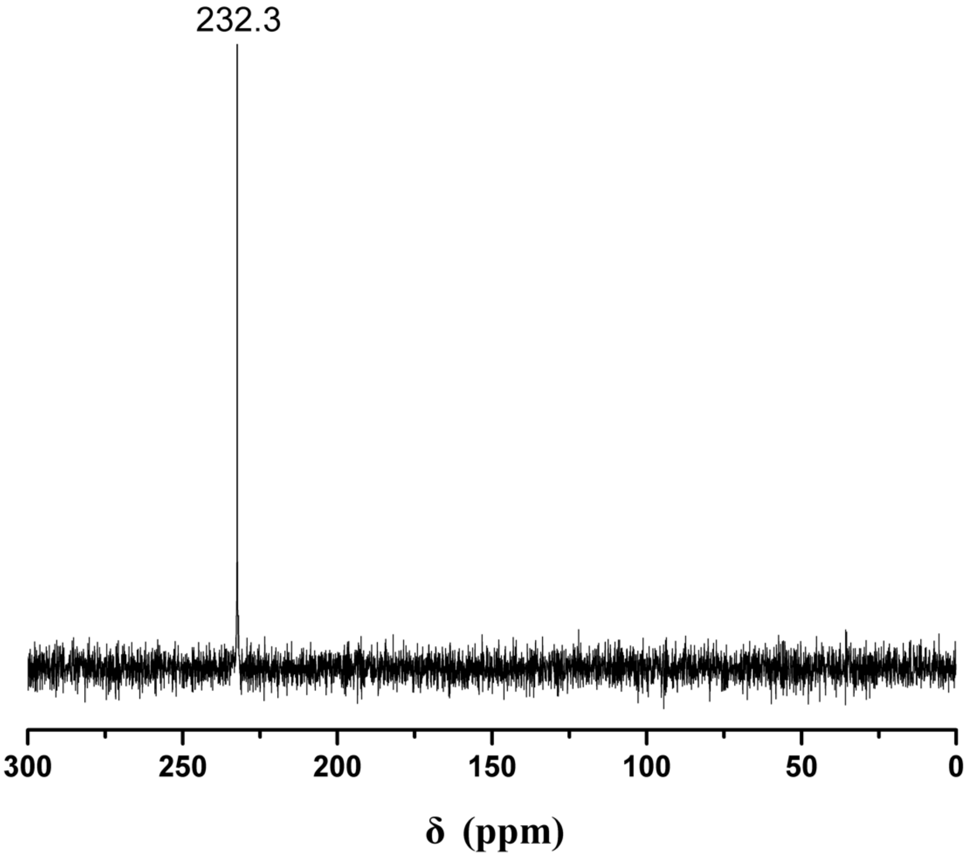

Figure S11. ^{45}Sc NMR spectrum of the reaction solution of Ga^{3+} & Sc^{3+} system recorded in H_2O at room temperature.

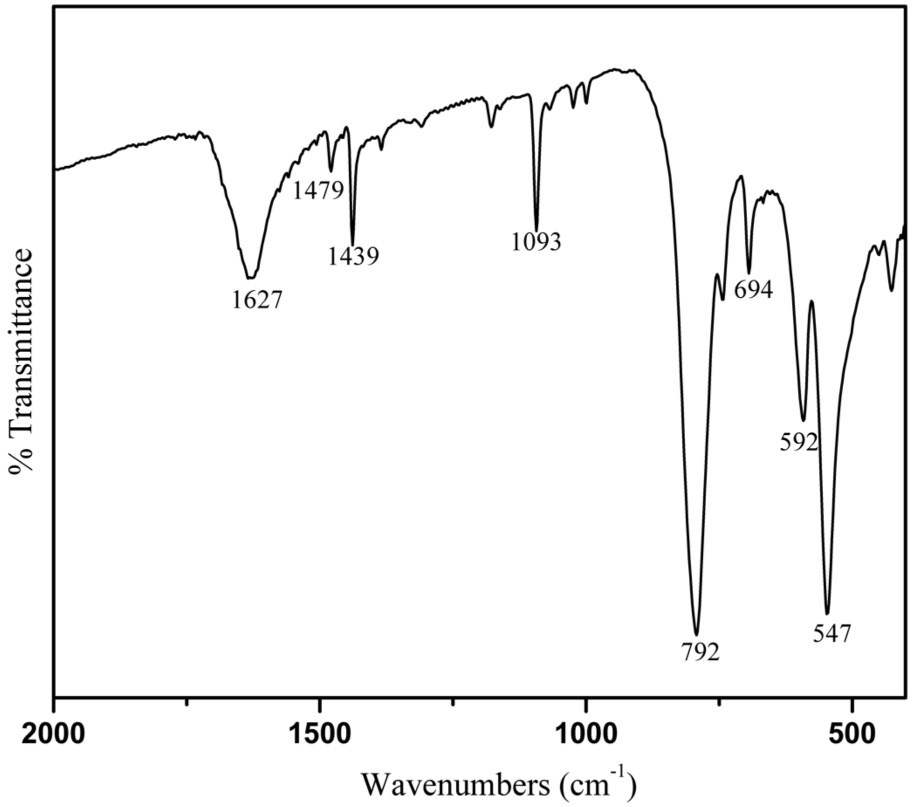

Figure S12. FT-IR spectrum of **Na-LaPd$_{12}$-closed**.

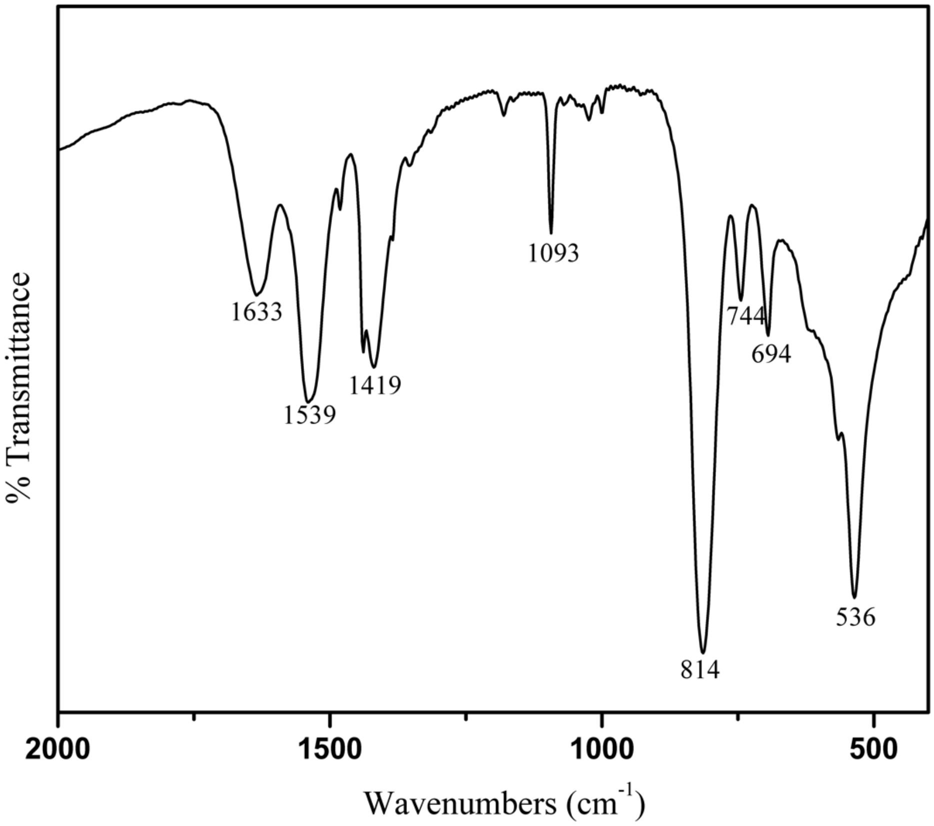

Figure S13. FT-IR spectrum of **Na-LaPd$_{12}$-open**.

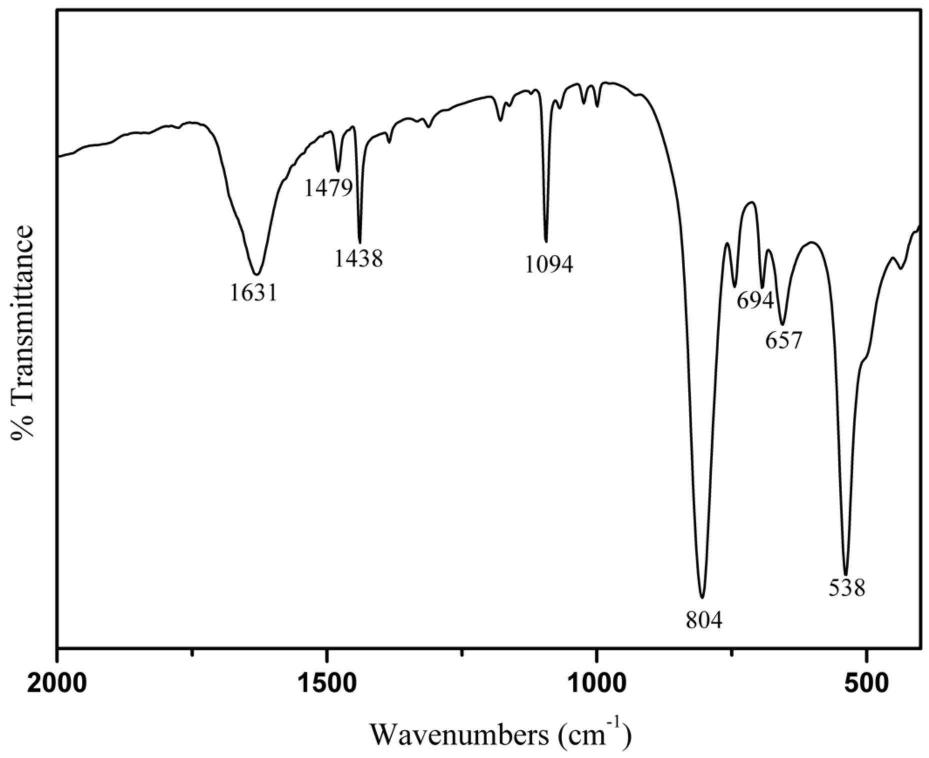

Figure S14. FT-IR spectrum of **Na-GaPd$_{12}$**.

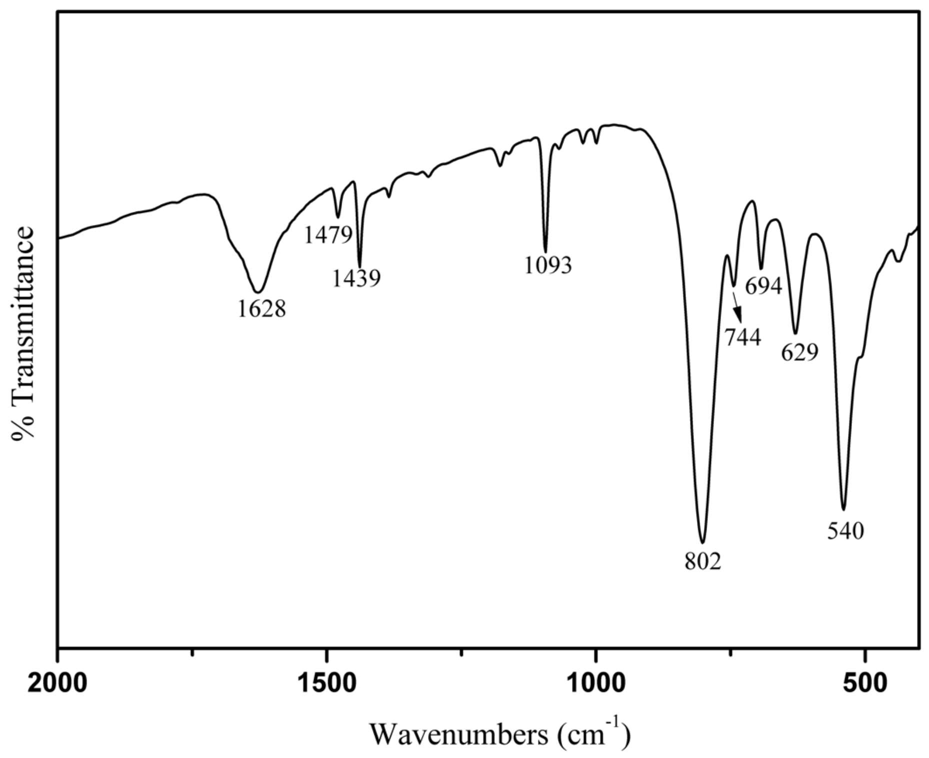

Figure S15. FT-IR spectrum of **Na-InPd$_{12}$**.

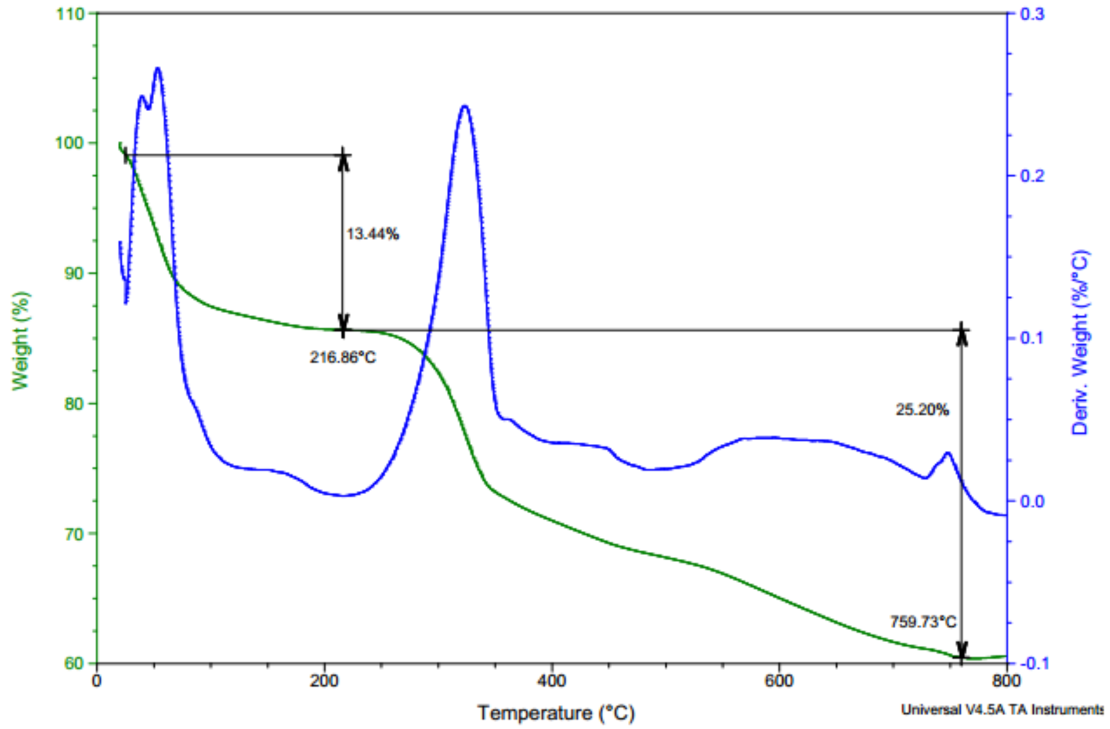

Figure S16. Thermogram of **Na-LaPd$_{12}$-closed** from 20 to 800 °C under N_2 atmosphere.

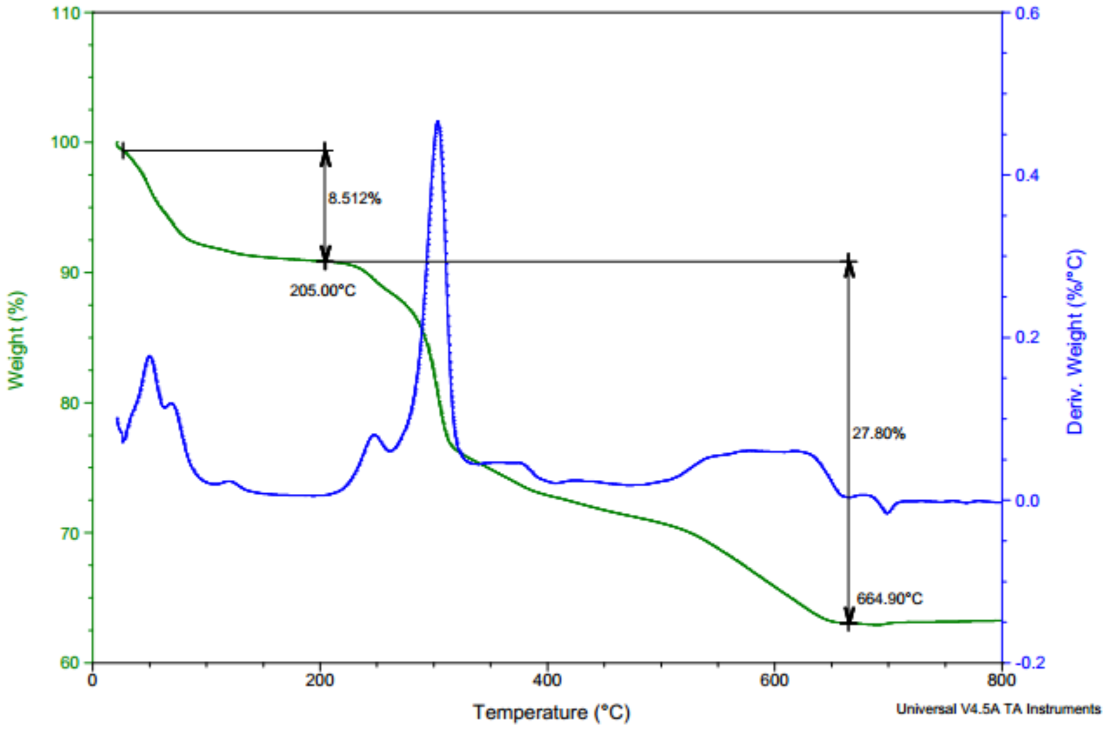

Figure S17. Thermogram of **Na-LaPd$_{12}$-open** from 20 to 800 °C under N_2 atmosphere.

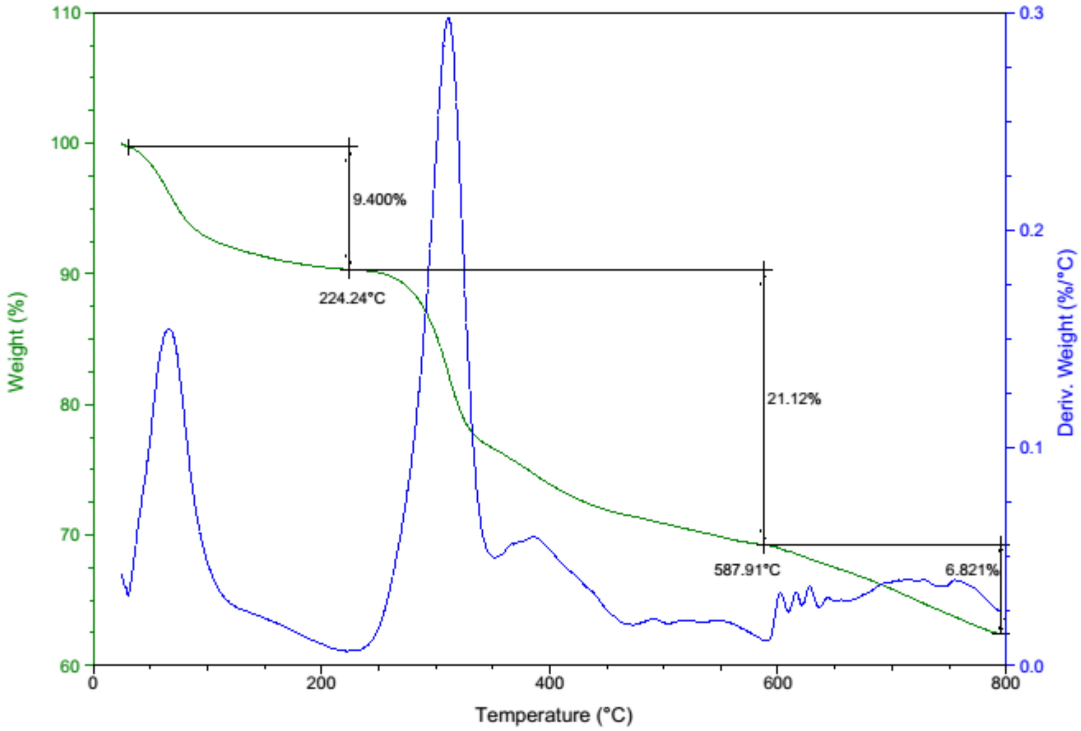

Figure S18. Thermogram of **Na-GaPd$_{12}$** from 20 to 800 °C under N_2 atmosphere.

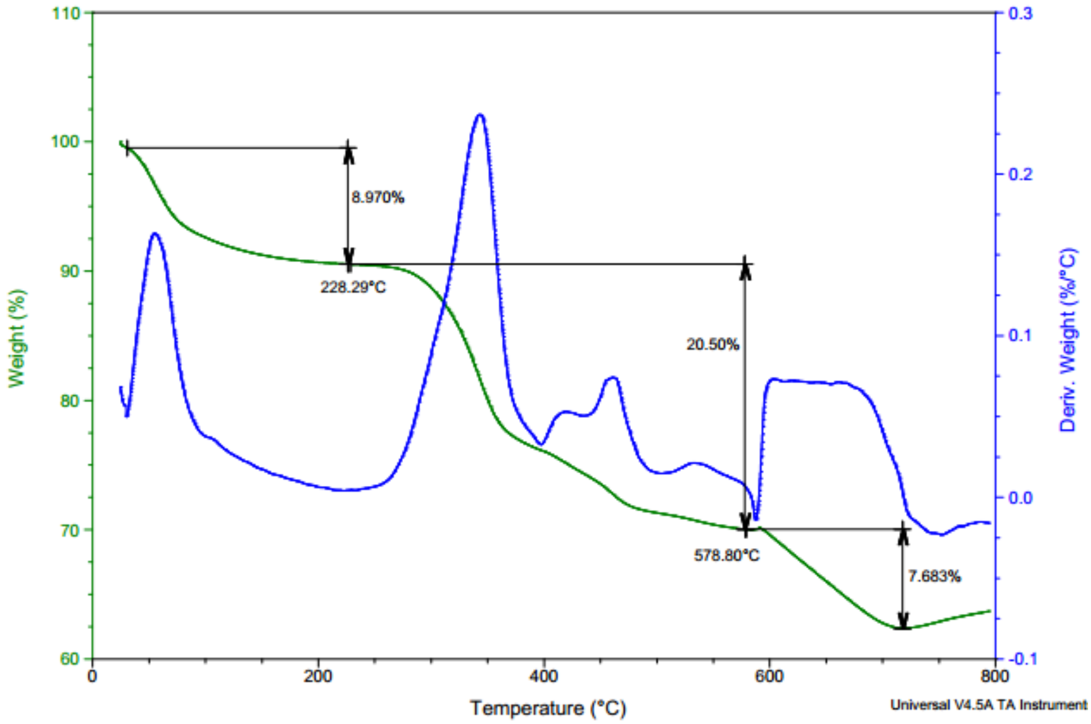

Figure S19. Thermogram of **Na-InPd$_{12}$** from 20 to 800 °C under N_2 atmosphere.
